# Supplementary material for: Inherited basis of visceral, abdominal subcutaneous and gluteofemoral fat depots
Source: Nat Commun. 2022 Jun 30;13:3771. doi: 10.1038/s41467-022-30931-2 (PMC9247093; doi:10.1038/s41467-022-30931-2)
Supplement: Supplementary file 1 — Supplementary Information [file 41467_2022_30931_MOESM1_ESM.pdf]

Inherited basis of visceral, abdominal subcutaneous and gluteofemoral fat depots

Agrawal*, Wang* et al.

Supplementary Methods

Convolutional Neural Networks to Compute VAT, ASAT, and GFAT Volumes

Supplementary Figure 1, **Supplementary Table 1**

Justification for BMI and Height adjustment for fat depot volumes

Supplementary Table 2

Quantifying extent of collider bias with BMI or height

Supplementary Figure 2-4, **Supplementary Table 3-5**

Supplementary Figure 5 Histograms for nine adiposity phenotypes

Supplementary Figure 6A-B Observational correlations between adiposity phenotypes and anthropometric measurements

Supplementary Figure 7A-B Genetic correlation between adiposity phenotypes and anthropometric measurements

Supplementary Figure 8-14 Manhattan plots

Supplementary Figure 15 Common variant sex heterogeneity for VAT/ASAT, VAT/GFAT, and ASAT/GFAT

Supplementary Figure 16-17 Cell-type enrichment analyses

Supplementary Figure 18 Visualizing the relationship between VATadj, ASATadj, and GFATadj and their polygenic scores at the tails of the distributions

Supplementary Table 6 Heritability of adiposity phenotypes

Supplementary Table 7 Nominally significant associations between the newly-identified adiposity loci in this study and cardiometabolic traits

Supplementary Table 8 Genomic inflation and LD-score intercepts

Supplementary Table 9 Genetic correlations between adiposity traits in males and females

Supplementary References

Supplementary Methods

Convolutional Neural Networks to Compute VAT, ASAT, and GFAT Volumes

A full description of the machine learning methods used to predict VAT, ASAT, and GFAT volumes including performance metrics and associations with type 2 diabetes and coronary artery disease is available in a prior manuscript.[1]

Among UK Biobank participants who underwent MRI imaging study, a subset had visceral adipose tissue (VAT) volume, abdominal subcutaneous adipose tissue (ASAT) volume, and total adipose tissue between the bottom of the thigh muscles to the top of vertebrae T9 (TAT) volume quantified and made available via the UK Biobank portal to the broader research community.[2–7] VAT (field 22407, "volume of the adipose tissue within the abdominal cavity, excluding adipose tissue outside the abdominal skeletal muscles and adipose tissue and lipids within and posterior of the spine and posterior of the back muscles") was available in 9,978 participants, ASAT (field 22408, "volume of the subcutaneous adipose tissue in the abdomen from the top of the femoral head to the top of the thoracic vertebrae T9") was available in 9,979, and TAT (field 22415, "total volume of adipose tissue, measured by MRI, between the bottom of the thigh muscles to the top of vertebrae T9") was available in 8,524. Based on these definitions, we additionally computed gluteofemoral adipose tissue (GFAT) volume:

GFAT = TAT (between top of T9 and bottom of thigh muscles) - VAT - ASAT

Given that the vast majority of adipose tissue between the top of vertebrae T9 and the top of the femoral head is accounted for by VAT or ASAT, GFAT was defined as total adipose tissue between the top of the femoral head and the bottom of the thigh muscles.

To train convolutional neural network models to measure VAT, ASAT, and GFAT, we first simplified the three-dimensional MRI images into composite two-dimensional projections of coronal and sagittal views, leading to an 830-fold reduction in data input size (Supplementary Figure 1). These machine learning models – trained on 80% of the participants with fat depots previously quantified – demonstrated near-perfect estimation association of each fat depot in the 20% of remaining individuals for each depot (r^2 = 0.991, 0.991, and 0.978 for VAT, ASAT, and GFAT, respectively).

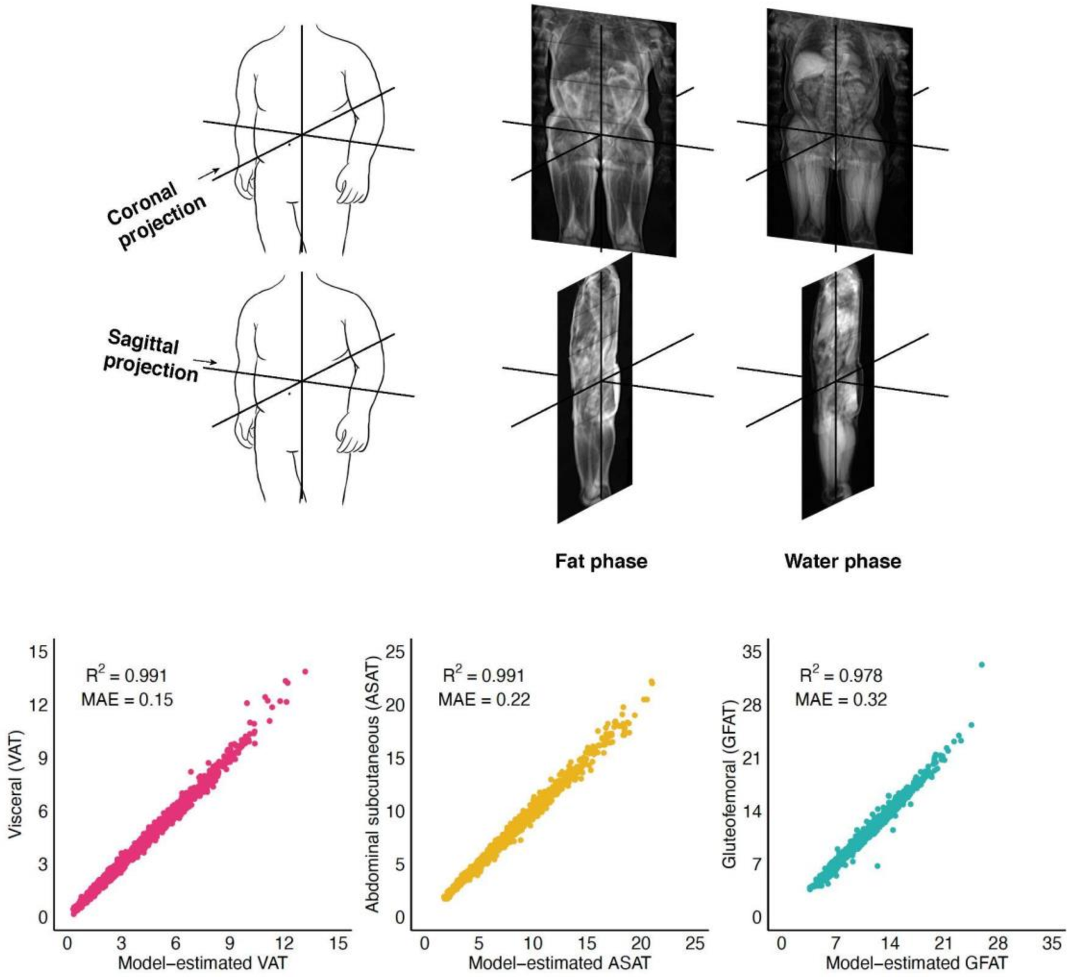

Supplementary Figure 1 Convolutional neural networks to quantify adipose tissue depots from body MRI images

(top row) Sample input into convolutional neural network (CNN): two-dimensional projections of MRIs in the coronal and sagittal directions with fat and water phases are used as input for each individual. (bottom row) In a 20% holdout set among each pre-labeled fat depot, the CNN achieves near-perfect prediction of that fat depot.

Finally, given that the gold standard for GFAT was derived from three other UK Biobank fields (VAT, ASAT, and TAT), we sought additional validation using DEXA-derived gynoid fat – corresponding to fat between the greater femoral trochanter and the mid-thigh – in UK Biobank. Among the 40,032 individuals with GFAT quantified from the above pipeline, 33,989 had gynoid fat mass available from DEXA imaging (multiplying gynoid total mass field 23265 and gynoid fat percent field 23264). Correlation between MRI-derived GFAT volume and DEXA-derived gynoid fat mass was very good (Pearson r = 0.96), supporting the validity of GFAT (Supplementary Table 1).

**Supplementary Table 1** Observational correlation between MRI-derived GFAT volume and DEXA-derived gynoid fat mass	
**Subgroup**	**Pearson correlation (r)**
Males	0.956
Females	0.962

Justification for BMI and Height adjustment for fat depot volumes

Initially motivated by seminal work on waist-hip ratio adjusted for BMI led by the GIANT consortium, we started by examining the properties of VAT, ASAT, and GFAT adjusted for BMI (but not height).[8] While genetic correlation with BMI was markedly reduced as desired, we noted that this adjustment introduced a significant genetic correlation with height (r_g ranging from 0.29 - 0.67) (Supplementary Table 2). As an example, GFAT adjusted for BMI (but not height) associated with rs67807996 ($P = 4.1 \times 10^{-14}$) and rs59985551 ($P = 2.1 \times 10^{-13}$) which have previously been identified as height-associated variants.[9,10]

A similar phenomenon has previously been noted with waist circumference adjusted for BMI (WCadjBMI) and hip circumference (HIPadjBMI) adjusted for BMI in work led by the GIANT consortium:

> "In contrast to WHRadjBMI, which has almost no genetic correlation with height ($r_g < 0.04$), WCadjBMI ($r_g = 0.42$) and HIPadjBMI ($r_g = 0.82$) have moderate genetic correlations with height. These data suggest that some, but not all, WCadjBMI and HIPadjBMI loci would be associated with height."[8]

Accordingly, one of the height-associated variants noted above – rs59985551 – has also been associated with WCadjBMI and HIPadjBMI.[11]

By additionally adjusting for height, VAT adjusted for BMI and height (VATadj), ASATadj, and GFATadj achieved near height-independence (r_g ranging from -0.04 - 0.02) as desired. This strategy is consistent with the goal of this study to nominate genetic variants associated with "local adiposity" – i.e. genetic variants that influence adipose tissue volume in specific fat depots independent of the "overall size" of an individual. Of note, adjustment of each fat depot for BMI and height led to values that were nearly identical – both in terms of observational and genetic correlation – to adjusting each fat depot for weight and height. This latter strategy has previously been used to adjust CT-derived pericardial fat prior to genetic association.[12,13]

Hence, our "adj" traits in this study are adjusted for BMI and height. More precisely, each adj trait represents residuals of sex-specific regressions of the fat depot of interest against age, age squared, BMI, and height.

**Supplementary Table 2** Genetic correlations between VAT, ASAT, and GFAT with various adjustment strategies and BMI and height		
	Genetic Correlation (r_g) with BMI	Genetic Correlation (r_g) with Height
VAT	0.663 (0.04)	0.104 (0.04)
ASAT	0.823 (0.02)	0.145 (0.04)
GFAT	0.692 (0.03)	0.367 (0.03)
VAT adjusted for BMI	-0.199 (0.06)	0.290 (0.04)
ASAT adjusted for BMI	-0.111 (0.05)	0.502 (0.03)
GFAT adjusted for BMI	-0.101 (0.05)	0.666 (0.03)
**VAT adjusted for BMI and Height**	**-0.165 (0.05)**	**-0.040 (0.05)**
**ASAT adjusted for BMI and Height**	**-0.068 (0.06)**	**0.018 (0.05)**
**GFAT adjusted for BMI and Height**	**-0.045 (0.05)**	**0.020 (0.04)**
VAT adjusted for Weight and Height	-0.176 (0.05)	-0.033 (0.04)
ASAT adjusted for Weight and Height	-0.077 (0.06)	0.027 (0.05)
GFAT adjusted for Weight and Height	-0.055 (0.05)	0.026 (0.04)
All genetic correlations are computed using LD-score regression as described in the Methods section of the manuscript.[14,15]		

Quantifying extent of collider bias with BMI or height

We determined that collider bias with BMI or height is minimally contributing to these results by conducting sensitivity analyses outlined in a recent large meta-analysis of WHRadjBMI[16]:

First, we determined the genome-wide genetic correlation between each of VATadj, ASATadj, and GFATadj with BMI and height, and compared to genetic correlations between WHRadjBMI and BMI and height (Supplementary Table 3). The greatest magnitude of genetic correlation was observed between

VATadj and BMI (r_g = -0.165, SE = 0.05) and this was comparable to the genetic correlation between WHRadjBMI and BMI (r_g = -0.109, SE = 0.07). Hence, from a genome-wide standpoint, the extent of collider bias with BMI and height was no more than that of WHRadjBMI.

Supplementary Table 3 Genetic correlations between VATadj, ASATadj, and GFATadj with BMI and height are comparable to those corresponding to WHRadjBMI

	Genetic Correlation (r_g) with BMI	Genetic Correlation (r_g) with Height
VAT adjusted for BMI and Height (VATadj)	-0.165 (0.05)	-0.040 (0.05)
ASAT adjusted for BMI and Height (ASATadj)	-0.068 (0.06)	0.018 (0.05)
GFAT adjusted for BMI and Height (GFATadj)	-0.045 (0.05)	0.020 (0.04)
WHRadjBMI	-0.109 (0.07)	-0.017 (0.05)

Genetic correlations between WHRadjBMI, BMI, and height are obtained using summary statistics from GWAS carried out in the same imaging cohort where analyses of VATadj, ASATadj, and GFATadj were done.

Next, we evaluated the fraction of lead SNPs ($P < 5 \times 10^{-8}$) for VATadj, ASATadj, and GFATadj that had stronger effect sizes for the unadjusted fat depot compared to effect sizes for BMI or height. We found that the majority of SNPs associated with adjusted fat depots were more strongly associated with the unadjusted fat depot than either of BMI or height (71-98%; Supplementary Table 4). For reference, 311/346 (90%) of the WHRadjBMI lead SNPs from a recent meta-analysis had a greater effect size magnitude for WHR than BMI.[16] This observation indicates that most genetic associations are unlikely to be secondary to collider bias with BMI or height.

**Supplementary Table 4** The majority of lead SNPs identified with VATadj, ASATadj, and GFATadj are more strongly associated with the unadjusted fat depot than BMI or height			
	Lead SNPs	Lead SNPs where effect size for unadjusted fat depot is greater than BMI effect size	Lead SNPs where effect size for unadjusted fat depot is greater than height effect size
VAT adjusted for BMI and Height (VATadj)	30	26 (87%)	24 (80%)
ASAT adjusted for BMI and Height (ASATadj)	21	18 (86%)	15 (71%)
GFAT adjusted for BMI and Height (GFATadj)	54	53 (98%)	52 (96%)

We additionally plotted each adjusted fat depot lead SNP on four plots to visualize data summarized in Supplementary Table 4 above (Supplementary Figure 2-4):

- Plot 1 (top left):
  - y-axis: -log10(P(unadjusted fat depot)/P(BMI))
  - x-axis: -log10(P(adjusted fat depot)
- Plot 2 (top right):
  - y-axis: beta(unadjusted fat depot)
  - x-axis: beta(BMI)
- Plot 3 (bottom left):
  - y-axis: -log10(P(unadjusted fat depot)/P(height))
  - x-axis: -log10(P(adjusted fat depot)
- Plot 4 (bottom right):
  - y-axis: beta(unadjusted fat depot)
  - x-axis: beta(height)

These plots are shown below:

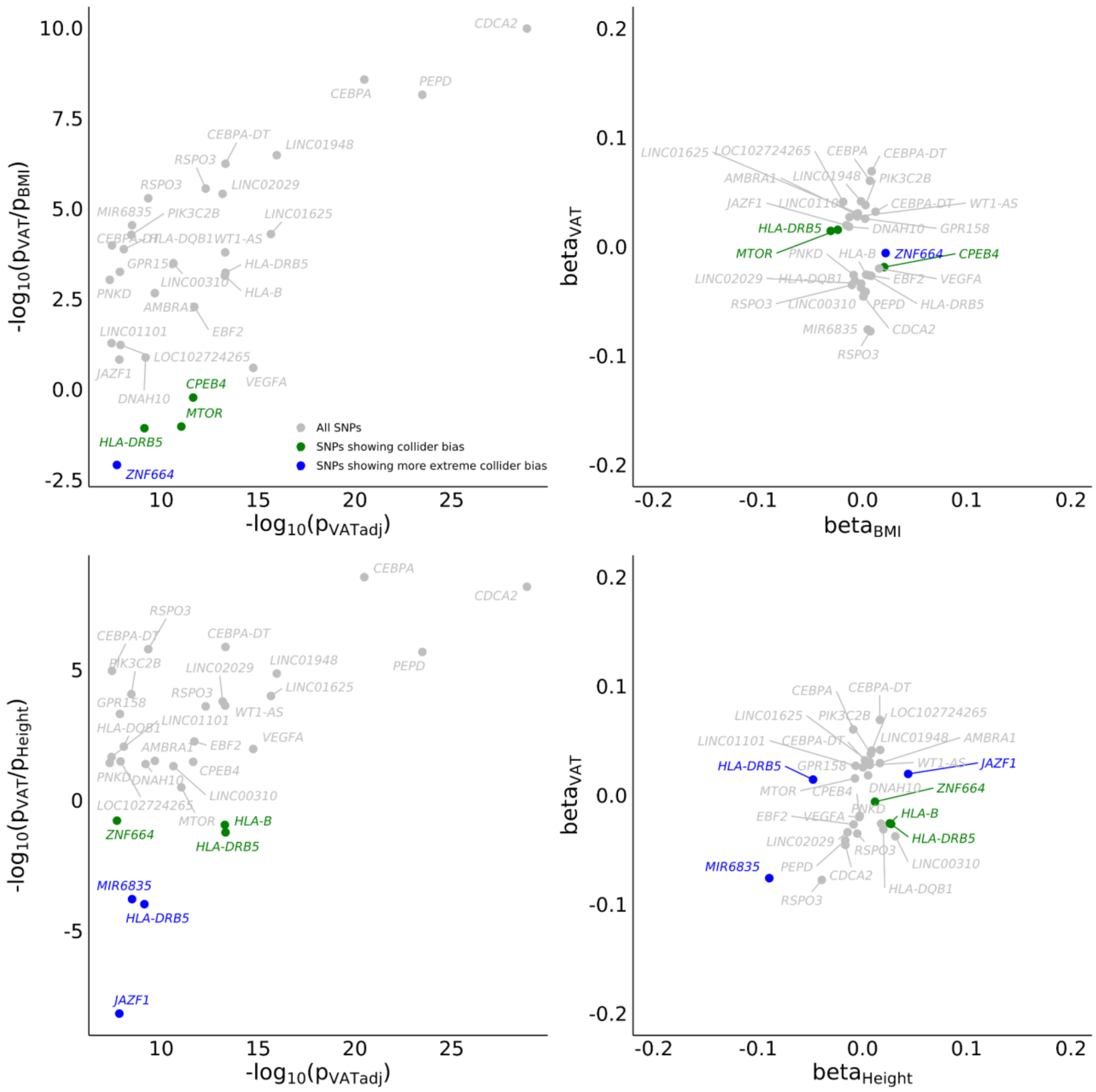

Supplementary Figure 2 Testing for VATadj collider bias with BMI and Height

(top row) Four of 30 VATadj lead SNPs are at risk of collider bias with BMI. (bottom row) Six of 30 VATadj lead SNPs are at risk of collider bias with height. SNPs showing collider bias are defined as -2 <= -log10(P_{VAT}/P_{BMI}) < 0, while extreme collider bias is defined as -log10(P_{VAT}/P_{BMI}) < -2. See Supplementary Data 22 for all data needed to plot these figures. P-values correspond to BOLT-LMM association P-values for each of the left panels.

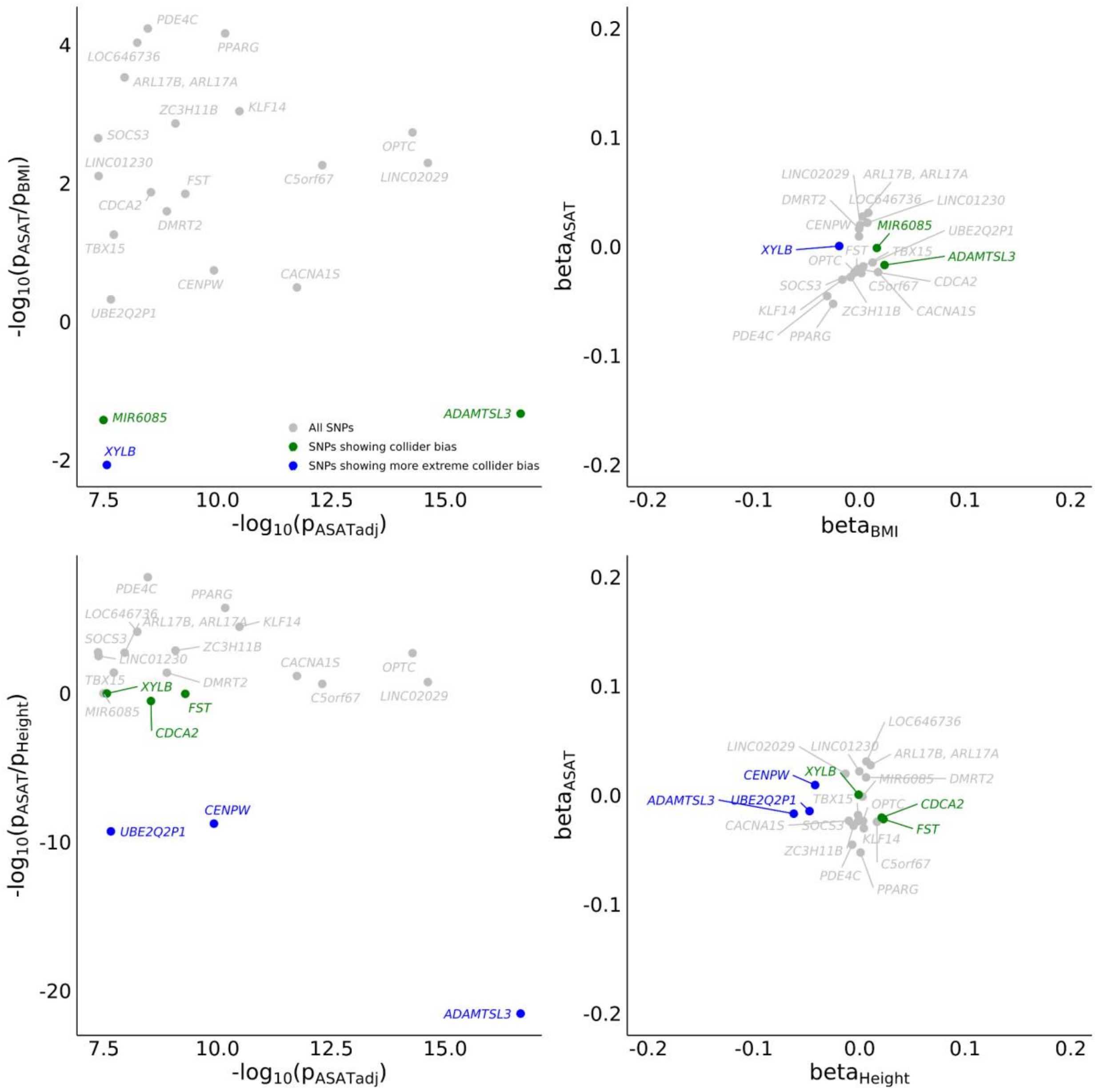

Supplementary Figure 3 Testing for ASATadj collider bias with BMI and Height

(top row) Three of 21 ASATadj lead SNPs are at risk of collider bias with BMI. (bottom row) Six of 21 ASATadj lead SNPs are at risk of collider bias with height. SNPs showing collider bias are defined as -2 <= -log10(P_{ASAT}/P_{BMI}) < 0, while extreme collider bias is defined as -log10(P_{ASAT}/P_{BMI}) < -2. See Supplementary Data 22 for all data needed to plot these figures. P-values correspond to BOLT-LMM association P-values for each of the left panels.

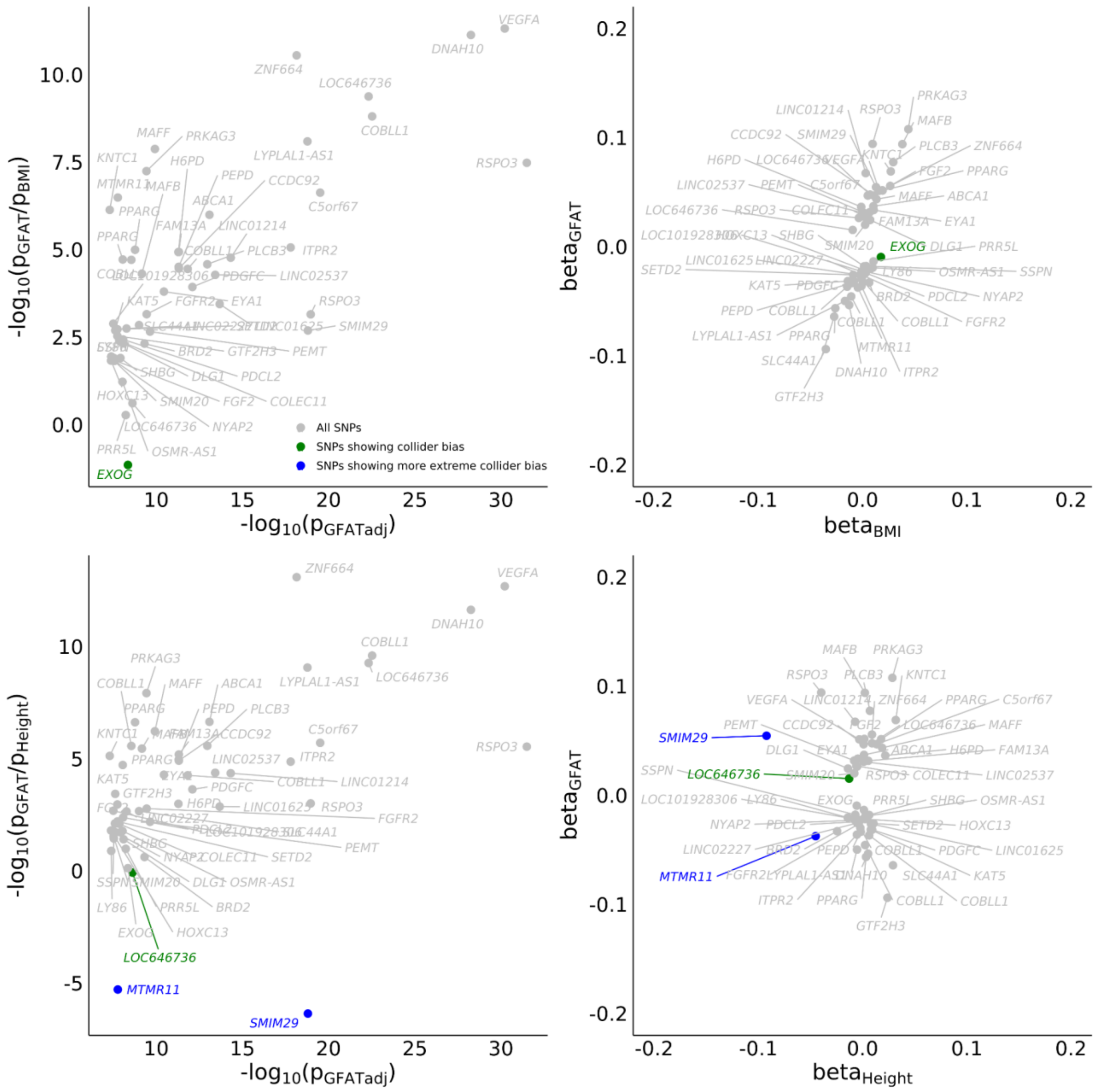

Supplementary Figure 4 Testing for GFATadj collider bias with BMI and Height

(top row) One of 54 GFATadj lead SNPs are at risk of collider bias with BMI. (bottom row) Two of 54 GFATadj lead SNPs are at risk of collider bias with height. SNPs showing collider bias are defined as -2 <= $-\log10(P_{GFAT}/P_{BMI}) < 0$, while extreme collider bias is defined as $-\log10(P_{GFAT}/P_{BMI}) < -2$. See Supplementary Data 22 for all data needed to plot these figures. P-values correspond to BOLT-LMM association P-values for each of the left panels.

Finally, we aimed to determine the effect of the VATadj, ASATadj, and GFATadj polygenic scores derived in this study on the corresponding metric, the corresponding unadjusted fat depot volume, BMI, and height. We found in each case that the polygenic score was significantly associated with the adjusted fat depot and the corresponding unadjusted fat depot, but not BMI or height (Supplementary Table 5). Taking GFATadj as an example, a 1-standard deviation increase in the polygenic score associated with increased GFATadj (beta = 0.27, P = 5.9e-122) and increased GFAT (beta = 0.15, P = 2.5e-38), but a null effect with BMI (beta = 0.02, P = 0.15) and height (beta = 0.02, P = 0.10).

Supplementary Table 5 Association of VATadj, ASATadj, and GFATadj polygenic scores with VATadj, ASATadj, GFATadj, unadjusted metrics, BMI, and height

PRS	Trait	Beta (95% CI)	P-value	Adjusted R^2
VATadj	VATadj	0.24 (0.22-0.26)	4.8e-101	0.0577
	VAT	0.13 (0.11-0.16)	4.8e-33	0.0179
	BMI	-0.02 (-0.04-0.01)	0.13	0.0001
	Height	-0.01 (-0.03-0.01)	0.54	0.0000
ASATadj	ASATadj	0.19 (0.17-0.21)	3.9e-62	0.0355
	ASAT	0.08 (0.06-0.11)	6.0e-14	0.0070
	BMI	0.00 (-0.02-0.02)	0.91	-0.0002
	Height	0.00 (-0.02-0.02)	0.78	-0.0001
GFATadj	GFATadj	0.27 (0.24-0.29)	5.9e-122	0.0703
	GFAT	0.15 (0.12-0.17)	2.5e-38	0.0210
	BMI	0.02 (-0.01-0.04)	0.15	0.0001
	Height	0.02 (0.00-0.04)	0.1	0.0003

Results reported here are from the 20% holdout set that was used to determine performance of polygenic scores. For all of VATadj, ASATadj, and GFATadj, the optimal set of LDpred2 hyperparameters in the validation set were p = 0.0056, h2 = 0.7, sparse = FALSE (Supplementary Table S22). To report performance metrics, each polygenic score was first adjusted for the first 10 PCs of genetic ancestry. Each PC-residualized polygenic score was then used to predict the trait of interest in a model that was adjusted for age at the time of imaging, sex, and the first 10 PCs of genetic ancestry. Betas correspond to sex-specific standard deviations per 1-standard deviation of the polygenic score. P-values correspond to the polygenic score term in each linear regression. The adjusted R2 corresponds to R2 of the full model minus R2 of a model containing only covariates.

In summary, our goal with the adjusted fat depot analyses was to understand the genetic architecture of "local adiposity" - i.e. adipose tissue volume in a given fat depot out of proportion to an individual's body size as captured by BMI and height. Sensitivity analyses above suggest:

- Adjusting for BMI + height avoids undesired genetic correlations with height that were previously noted for WCadjBMI and HIPadjBMI[8]; of note, adjustment for BMI + height is nearly identical to adjustment for weight + height, which was employed previously to adjust CT-derived pericardial fat prior to genetic association[12,13]
- Carrying out sensitivity analyses to determine the extent of collider bias as outlined by Pulit et al. for WHRadjBMI[16], we determine that collider bias with BMI or height is unlikely to be driving the majority of the discovered associations for VATadj, ASATadj, and GFATadj

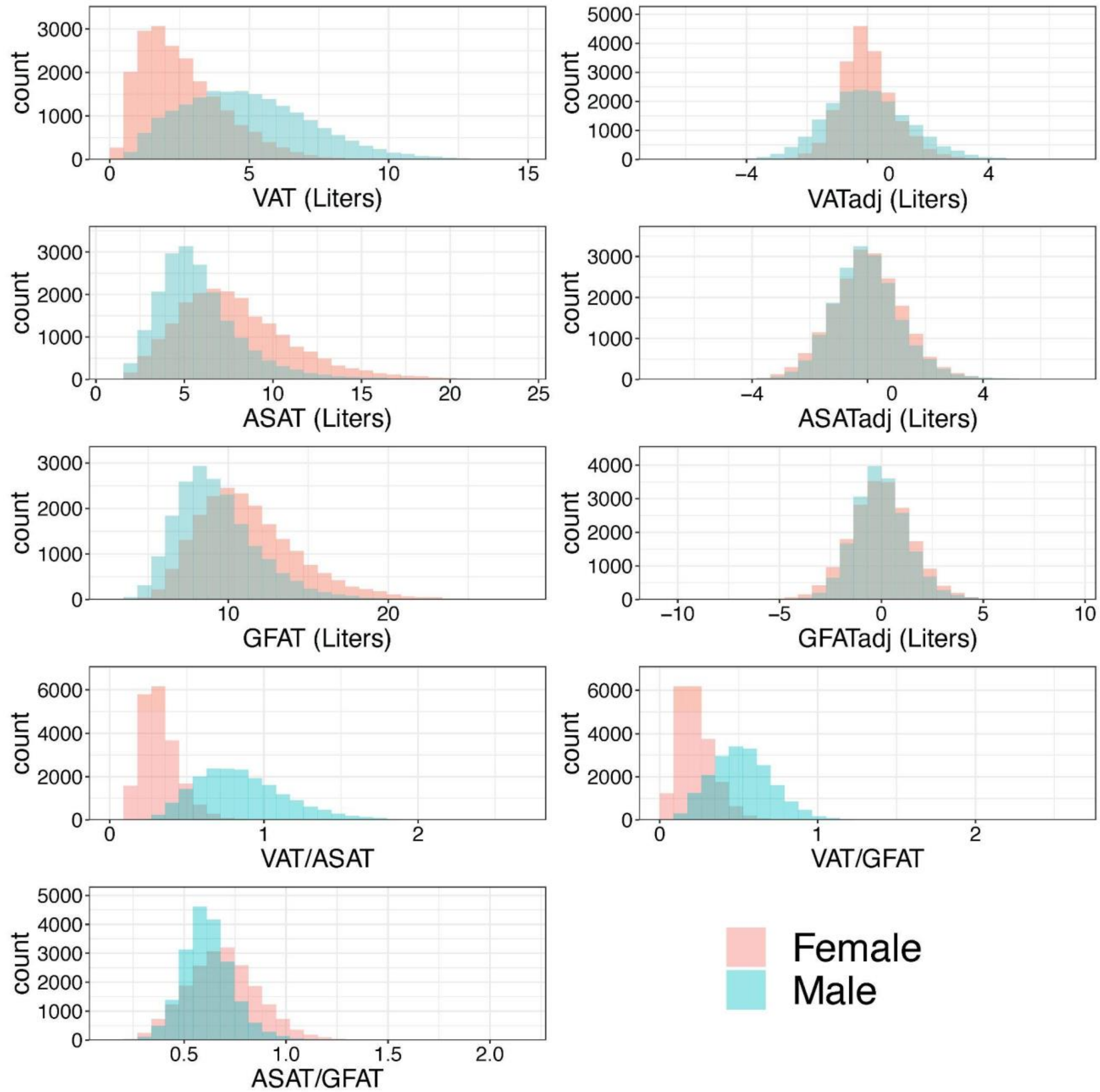

Supplementary Figure 5 Histograms for nine adiposity phenotypes

Individuals who passed imaging quality control and have been genotyped (Supplementary Data 1, n = 39,076) are plotted here in a sex-stratified fashion. Note that BMI was unavailable in 1,326 (3%) of individuals, so 37,750 individuals are plotted for VATadj, ASATadj, and GFATadj. Note that sex-specific residuals prior to any additional normalization are plotted for VATadj, ASATadj, and GFATadj.

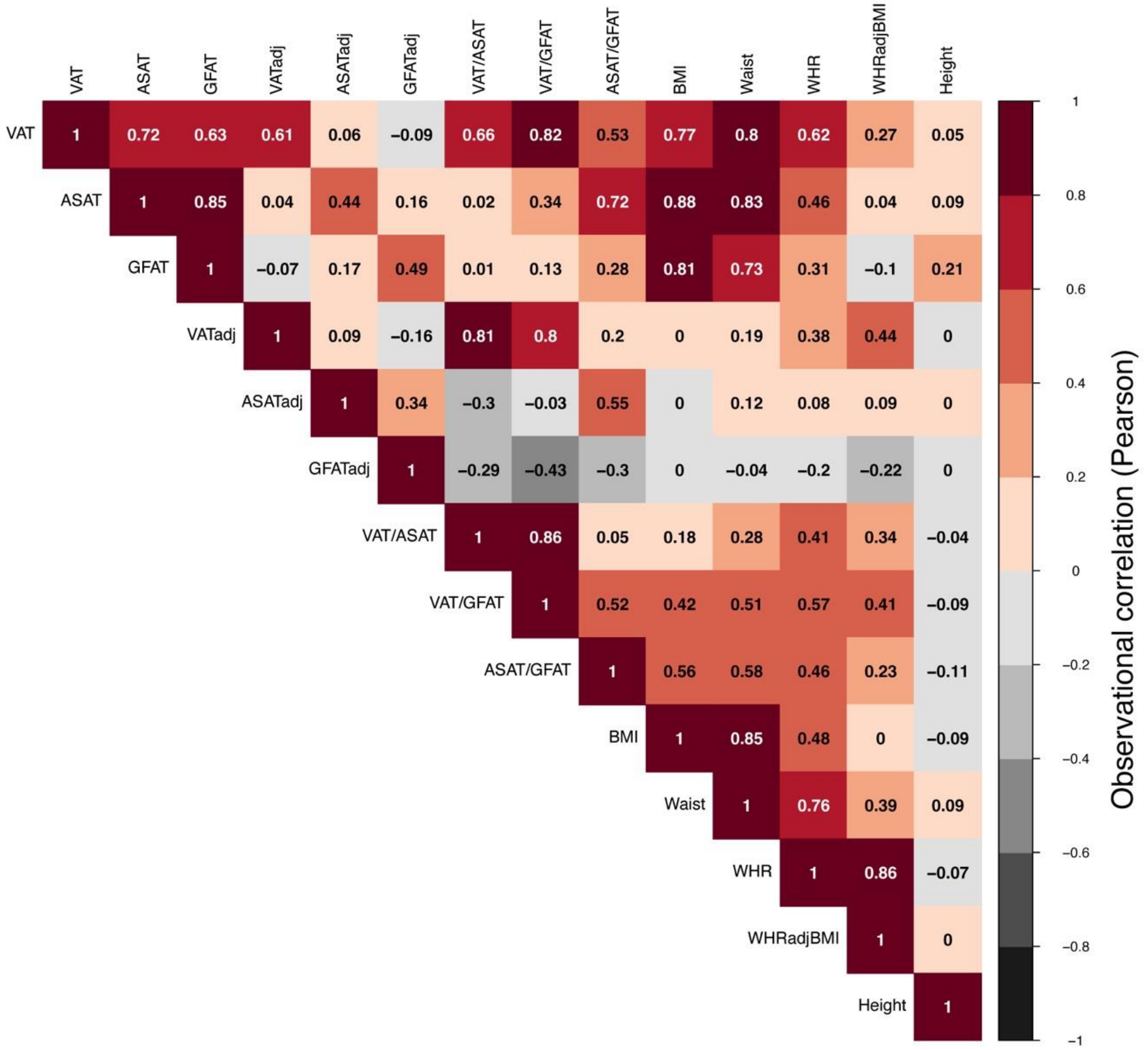

Supplementary Figure 6A Observational correlations between adiposity phenotypes and anthropometric measurements (sex-combined)

Pearson correlation coefficients between 9 adiposity traits and 5 anthropometric measures are shown. Each phenotype was scaled to mean 0 and variance 1 in sex-stratified groups prior to computing the Pearson correlation.

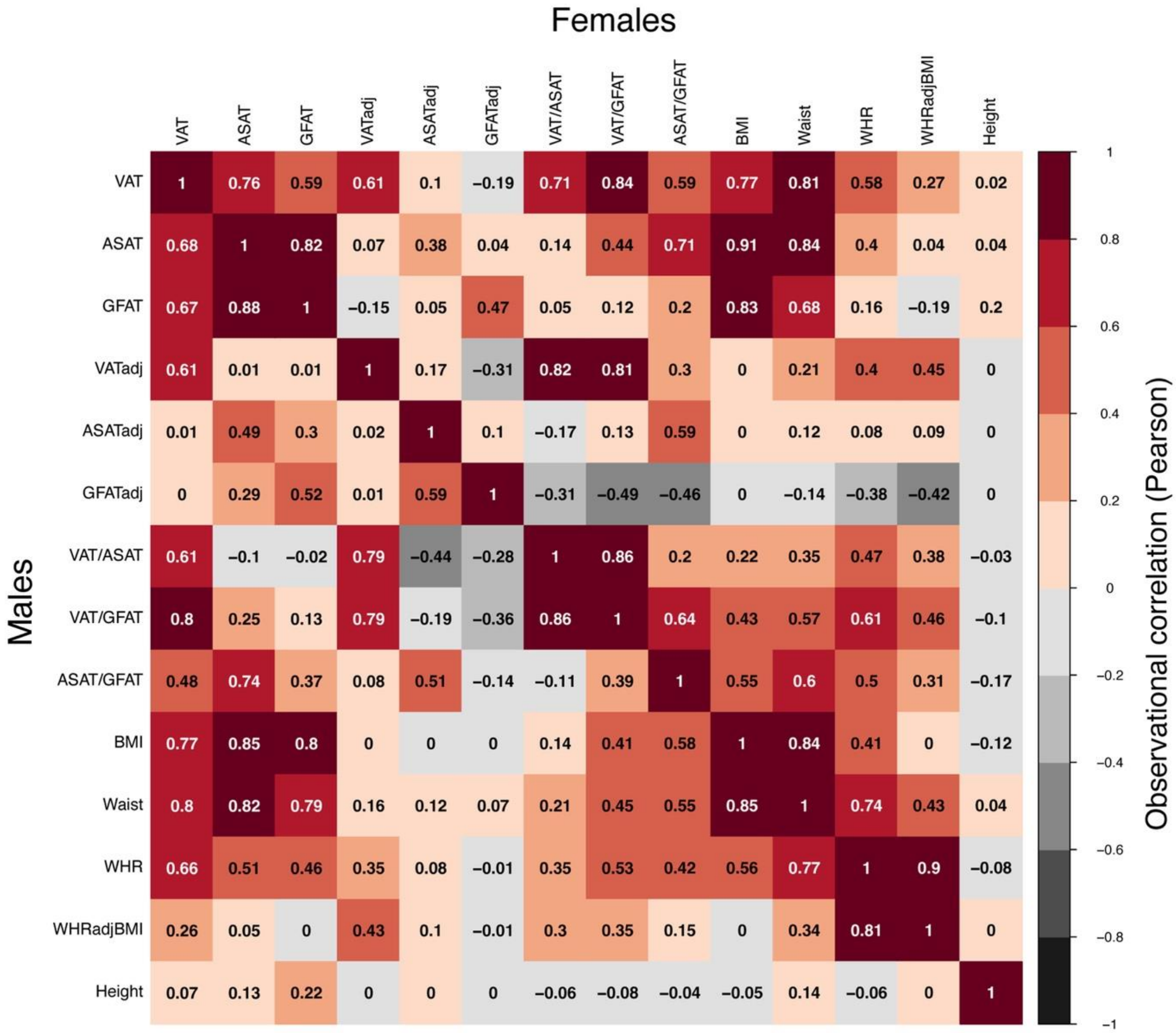

Supplementary Figure 6B Observational correlations between adiposity phenotypes and anthropometric measurements (sex-stratified)

Sex-stratified Pearson correlation coefficients between 9 adiposity traits and 5 anthropometric measures are shown.

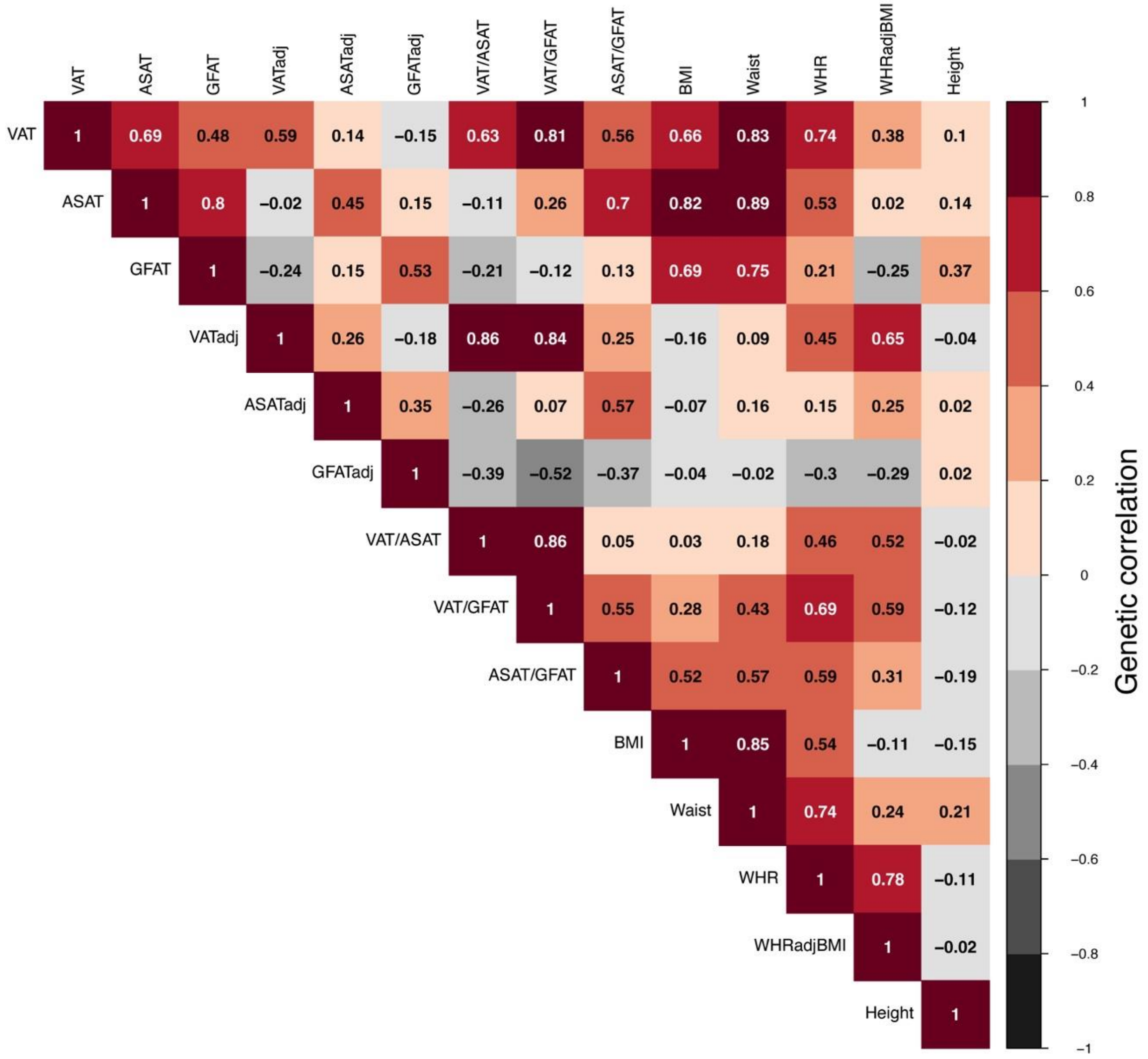

Supplementary Figure 7A Genetic correlation between adiposity phenotypes and anthropometric measurements (sex-combined)

Genetic correlations (r_g) between 9 adiposity traits and 5 anthropometric measures were estimated from cross-trait LD-score regression using summary statistics from sex-combined GWAS of these traits in UK Biobank.[14]

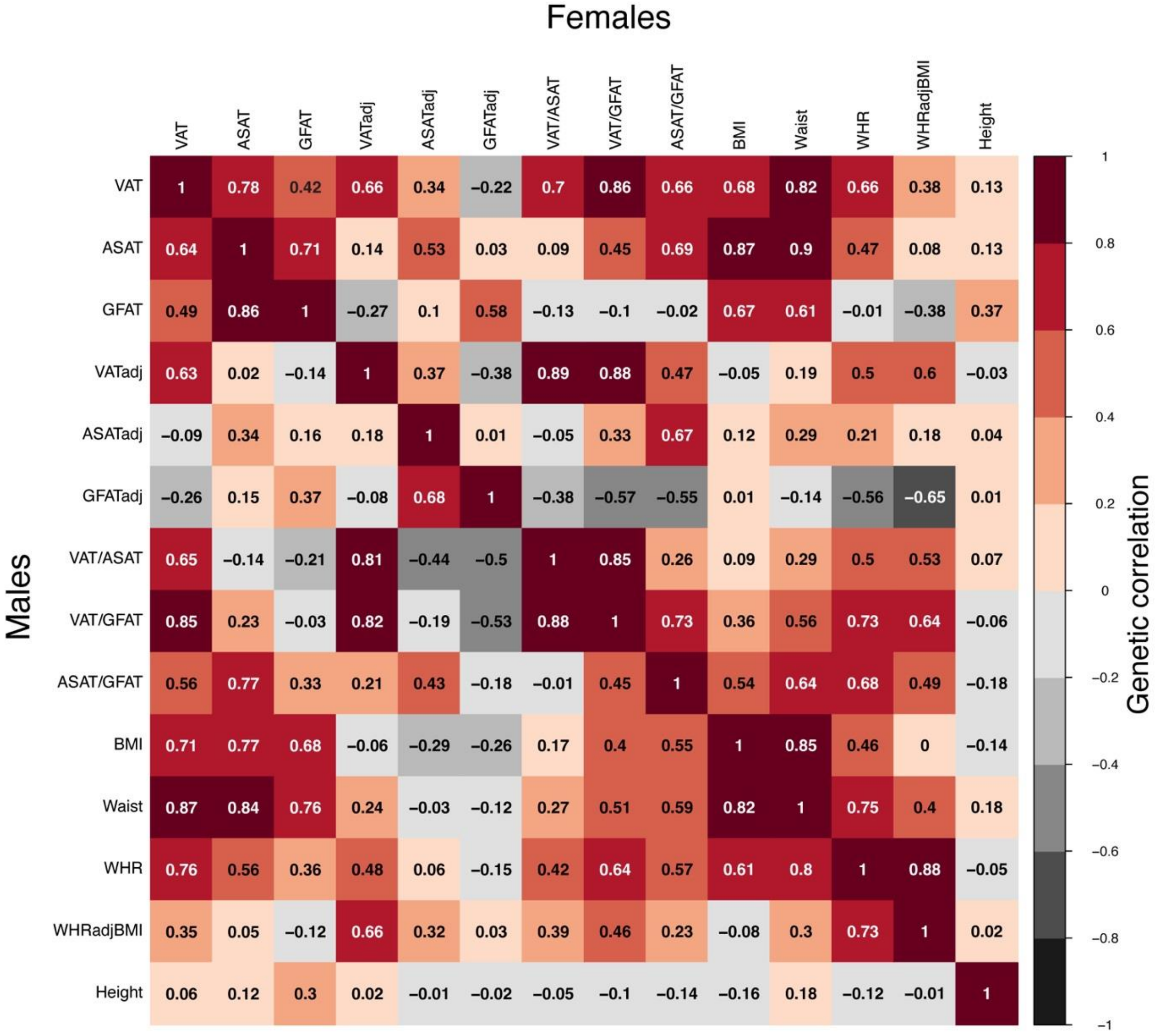

Supplementary Figure 7B Genetic correlation between adiposity phenotypes and anthropometric measurements (sex-stratified)

Genetic correlations (r_g) estimated with cross-trait LD-score regression using summary statistics from sex-stratified GWAS of these traits in UK Biobank.

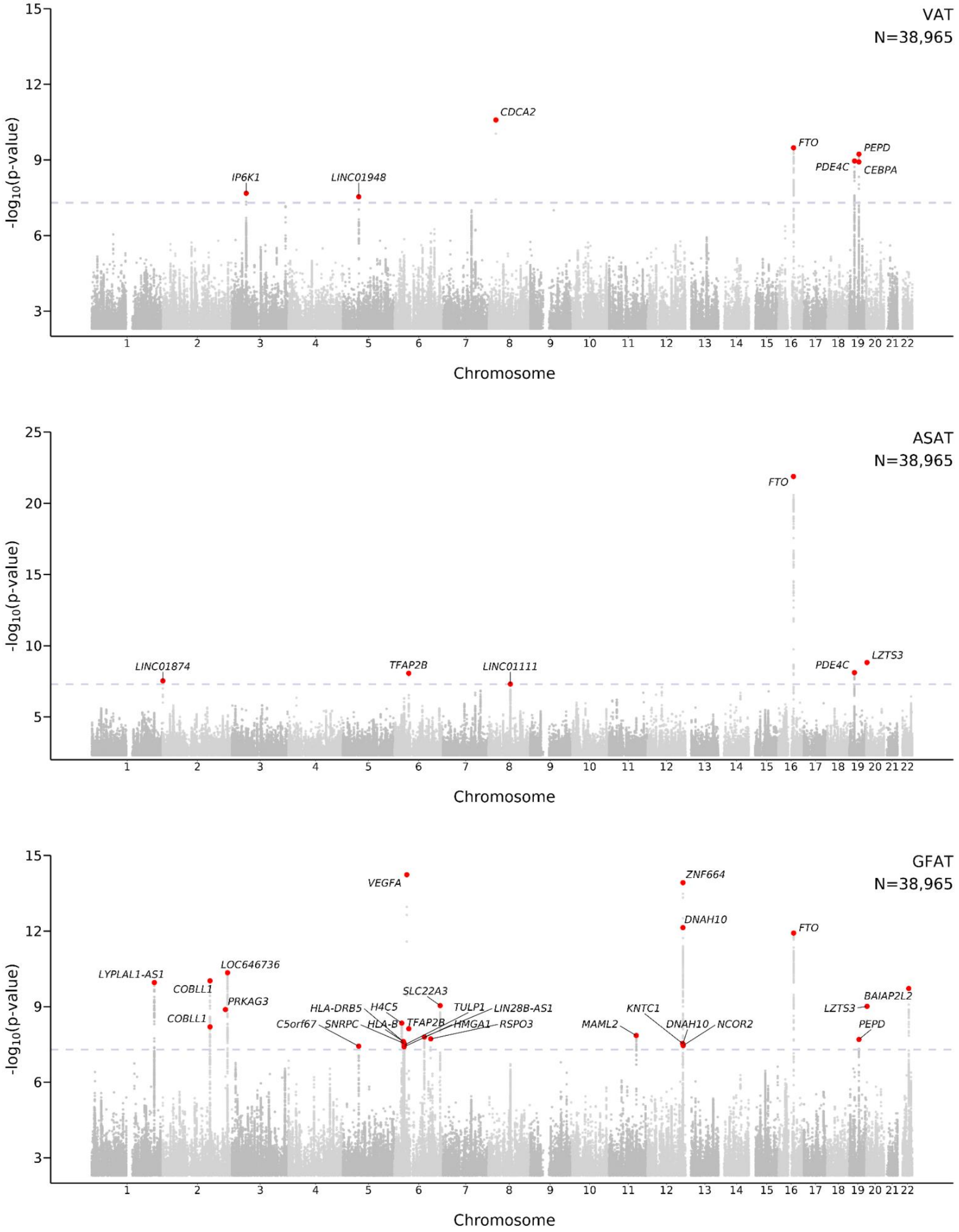

Supplementary Figure 8 Manhattan plots of unadjusted VAT, ASAT, and GFAT volumes

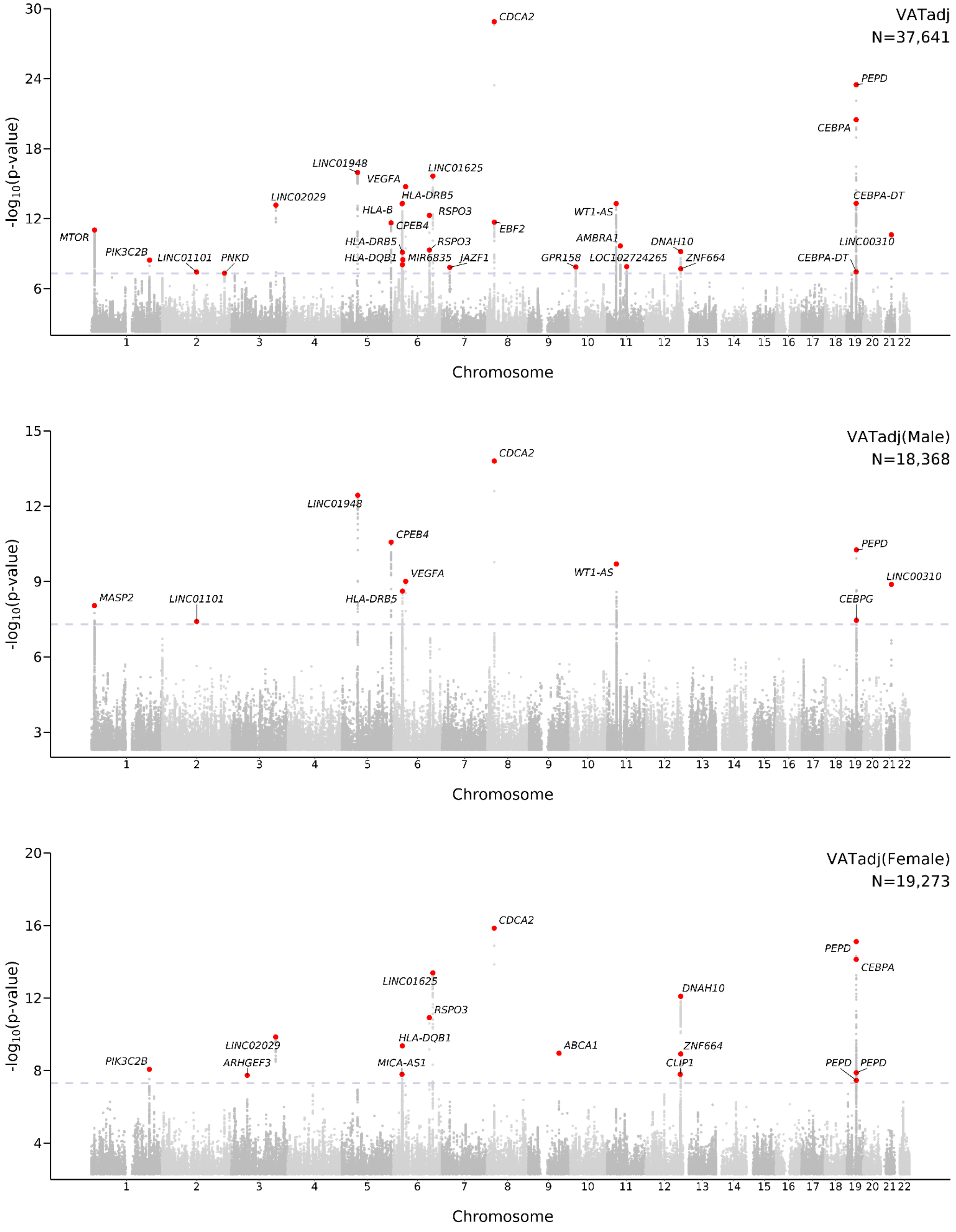

Supplementary Figure 9 Manhattan plots of VATadj (sex-combined and sex-stratified)

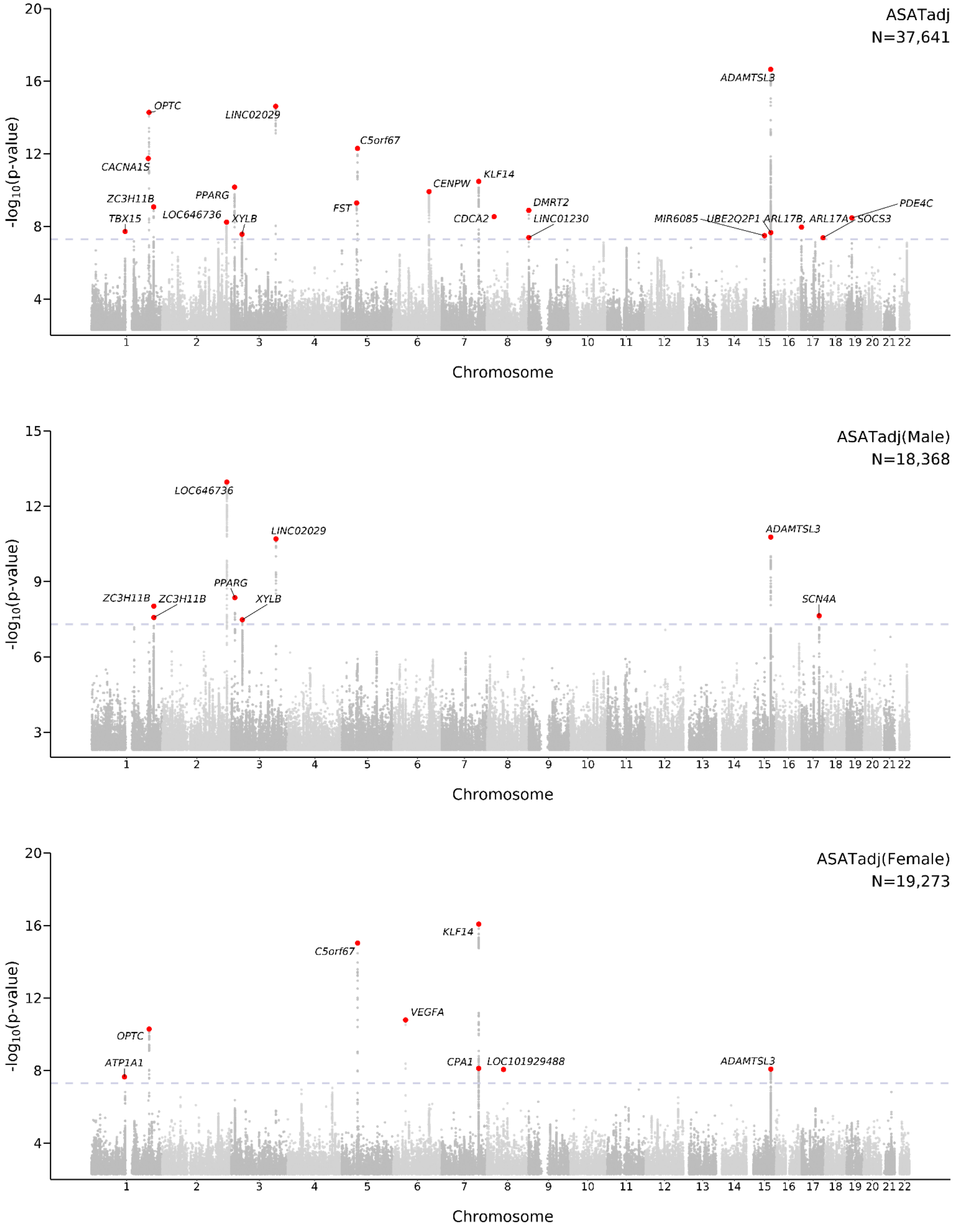

Supplementary Figure 10 Manhattan plots of ASATadj (sex-combined and sex-stratified)

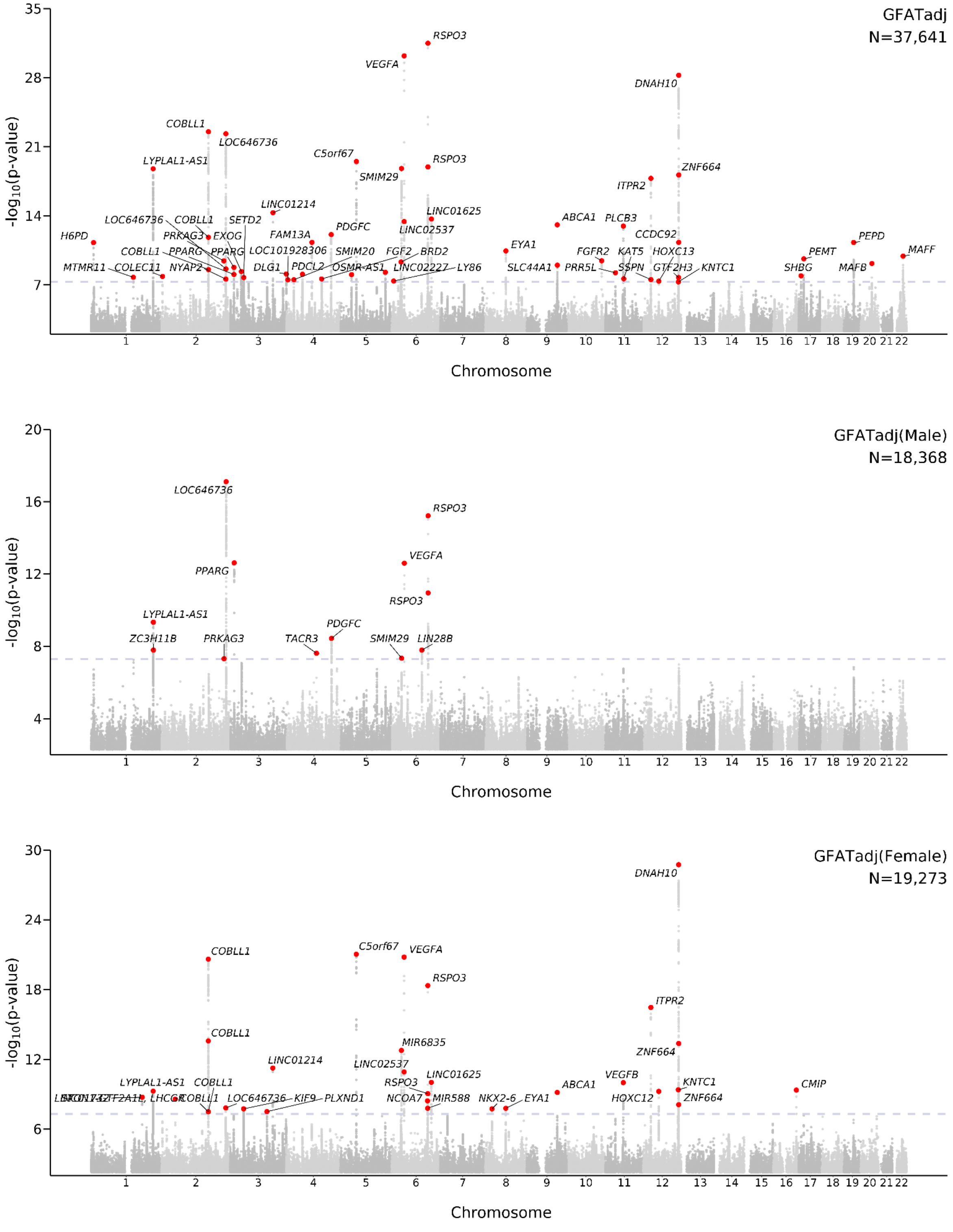

Supplementary Figure 11 Manhattan plots of GFATadj (sex-combined and sex-stratified)

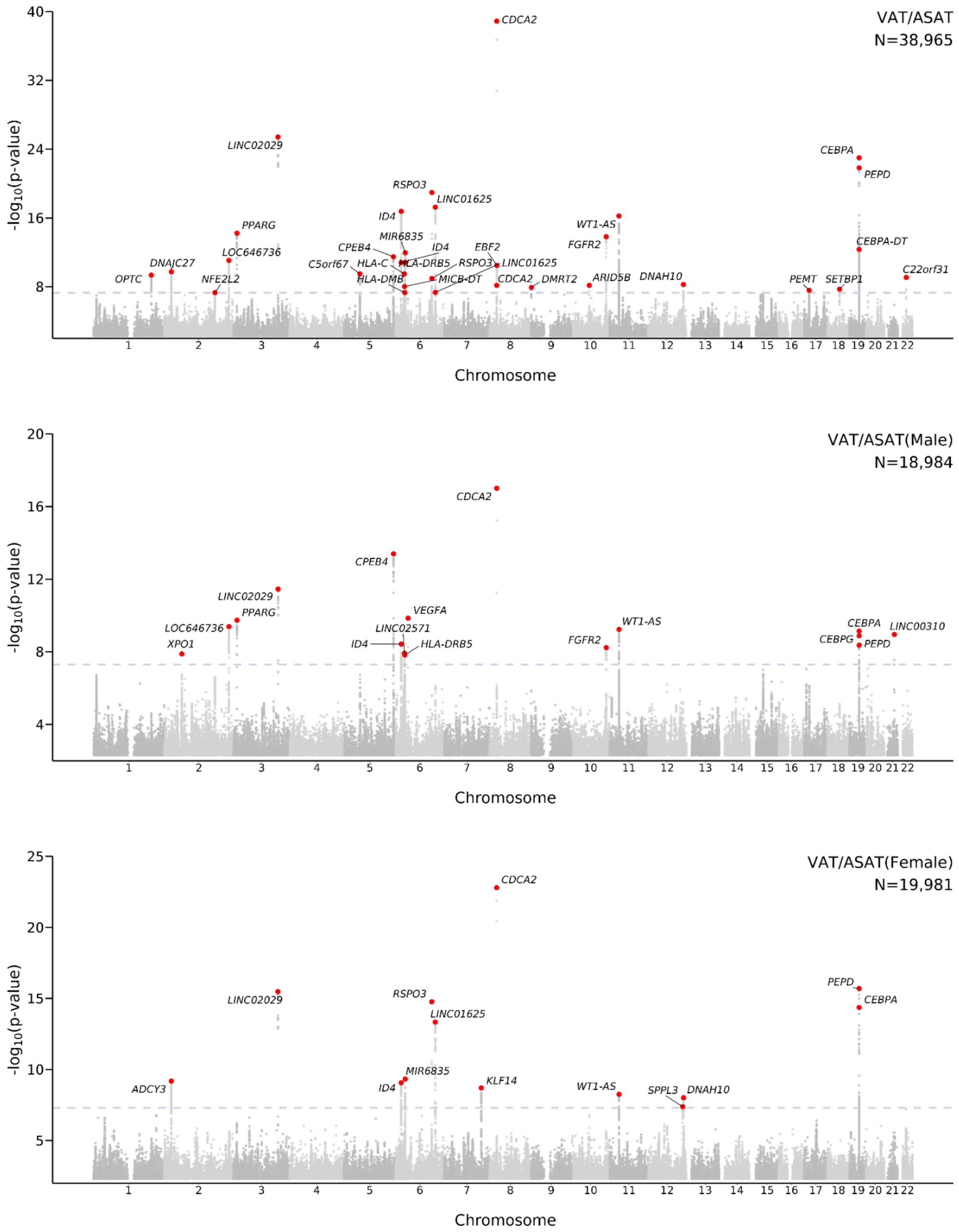

Supplementary Figure 12 Manhattan plots of VAT/ASAT ratio (sex-combined and sex-stratified)

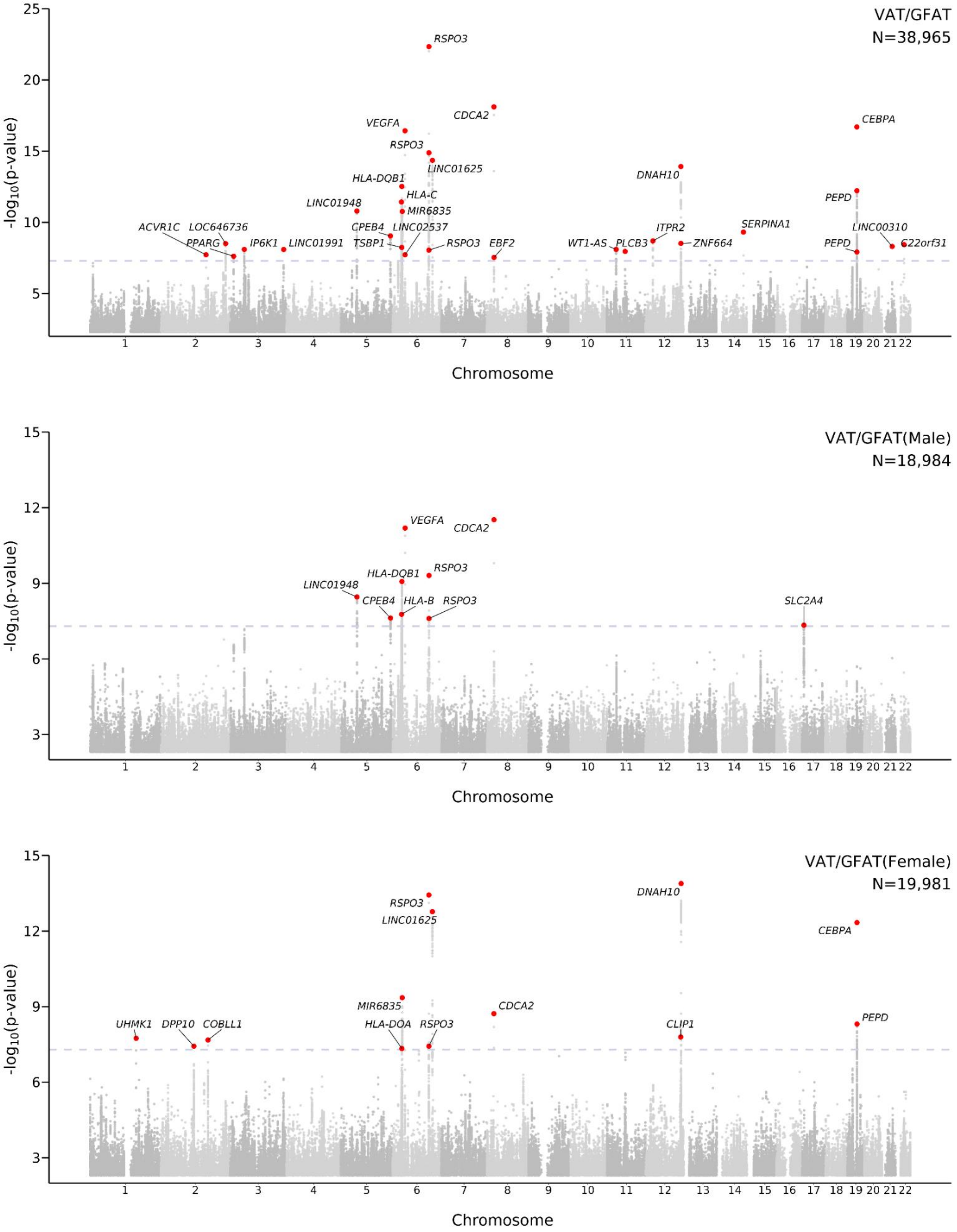

Supplementary Figure 13 Manhattan plots of VAT/GFAT ratio (sex-combined and sex-stratified)

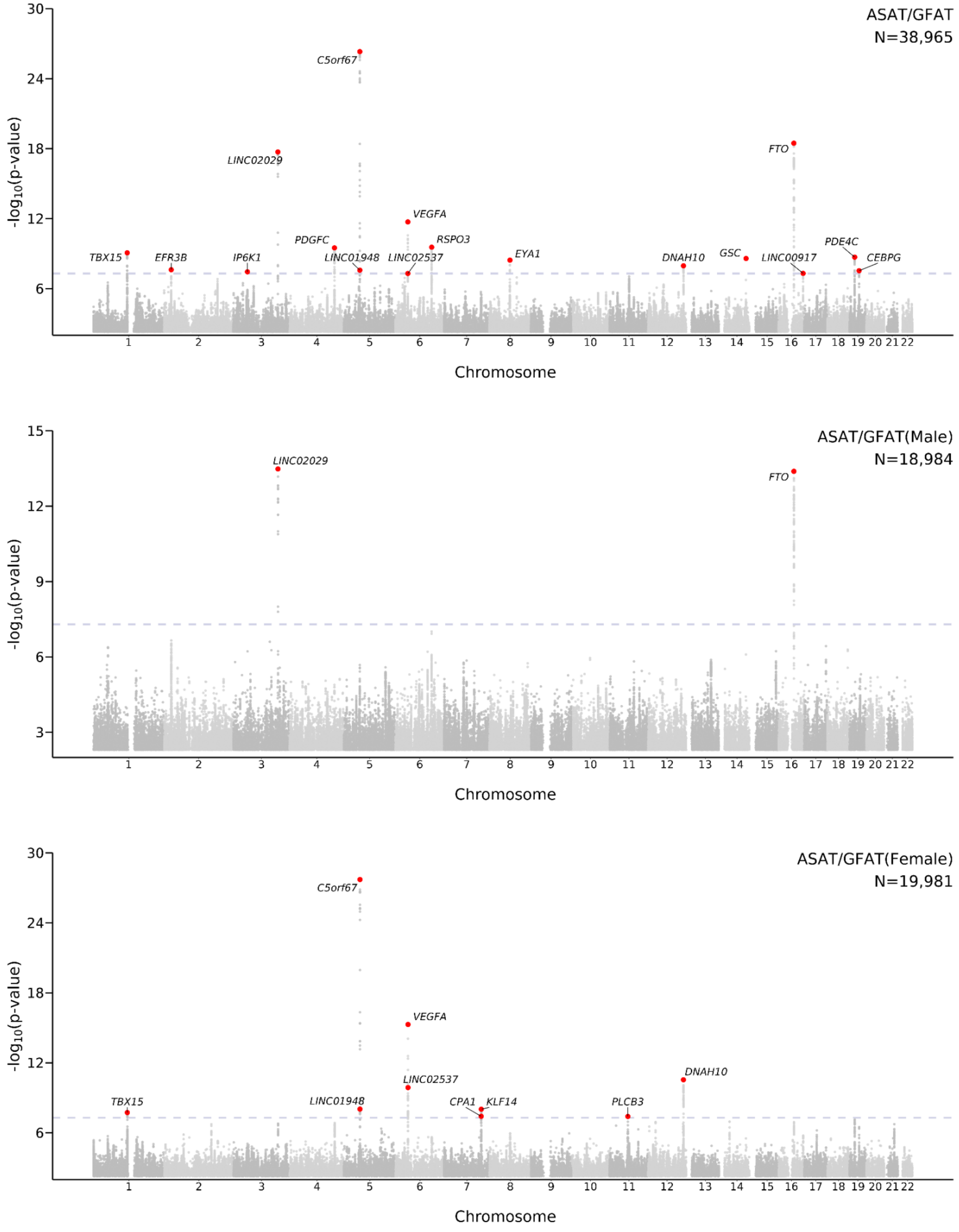

Supplementary Figure 14 Manhattan plots of ASAT/GFAT ratio (sex-combined and sex-stratified)

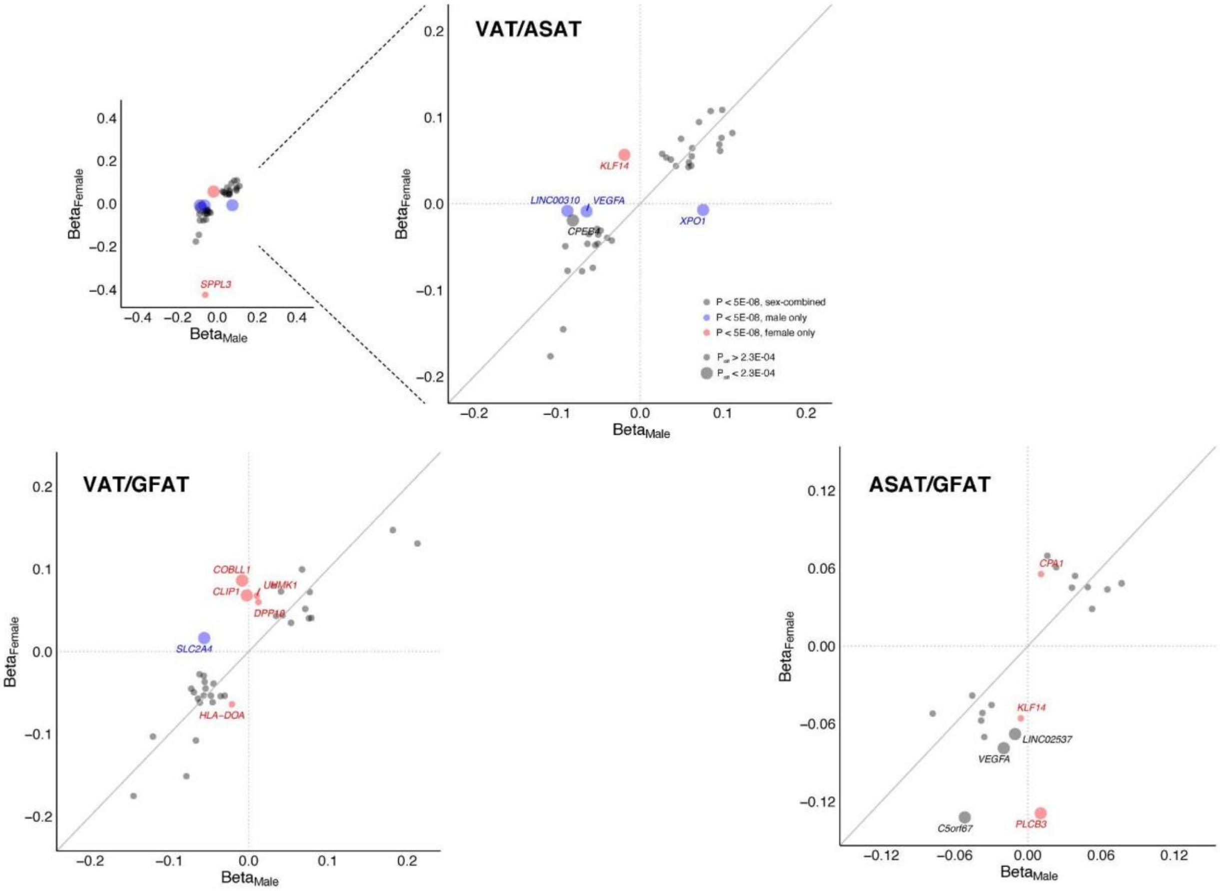

Supplementary Figure 15 Common variant sex heterogeneity for VAT/ASAT, VAT/GFAT, and ASAT/GFAT

For each adiposity trait, independent loci that were associated with the trait in either sex-combined or sex-stratified analyses are plotted (Supplementary Data 10). 38 such loci are plotted for VAT/ASAT, 36 for VAT/GFAT, and 20 for ASAT/GFAT. Black loci were genome-wide significant (P < 5E-08) in sex-combined analysis, blue loci were significant for males, but neither females nor sex-combined, and red loci were significant for females, but neither males nor sex-combined. P_{diff} indicates the P-value for a hypothesis test comparing SNP effects in males and females, as implemented in EasyStrata software (Methods). Across six adiposity traits (VATadj, ASATadj, GFATadj, VAT/ASAT, VAT/GFAT, and ASAT/GFAT), 220 unique loci-trait pairs were tested for sex heterogeneity, so a significance threshold of $P_{diff} < 0.05/220 = 2.3 \times 10^{-4}$ was set – large circles indicate that a given locus met this criterion.

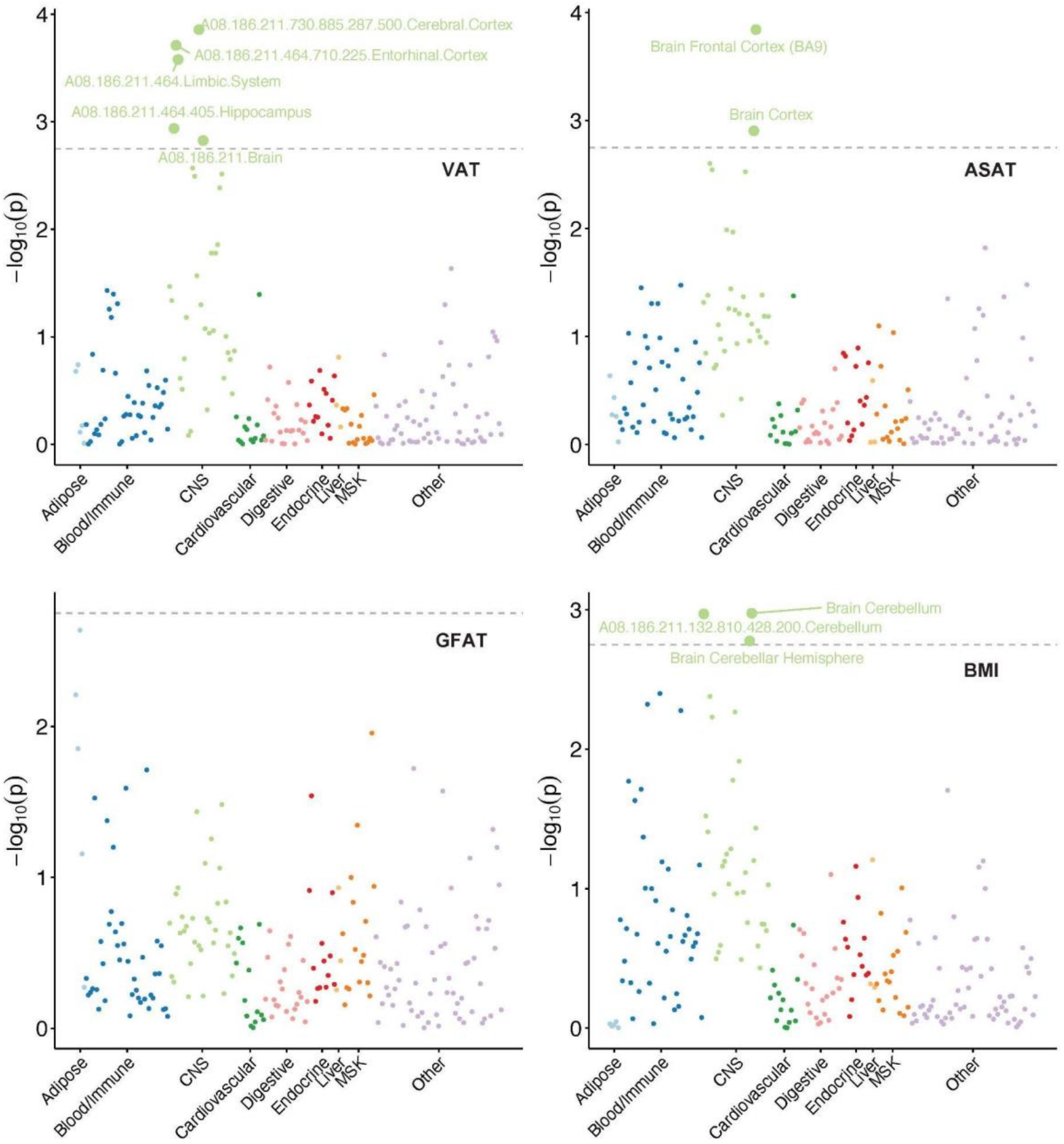

Supplementary Figure 16 Cell-type enrichment for VAT, ASAT, GFAT, and BMI

Top left: VAT; Top right: ASAT, Bottom left: GFAT, Bottom right: BMI. Each circle represents a tissue or cell type from either the GTEx dataset or the Franke lab dataset. Large circles pass the cutoff of FDR < 5% at –log10 (P) = 2.75.[17] Complete data tables corresponding to these plots are found in Supplementary Data 14.

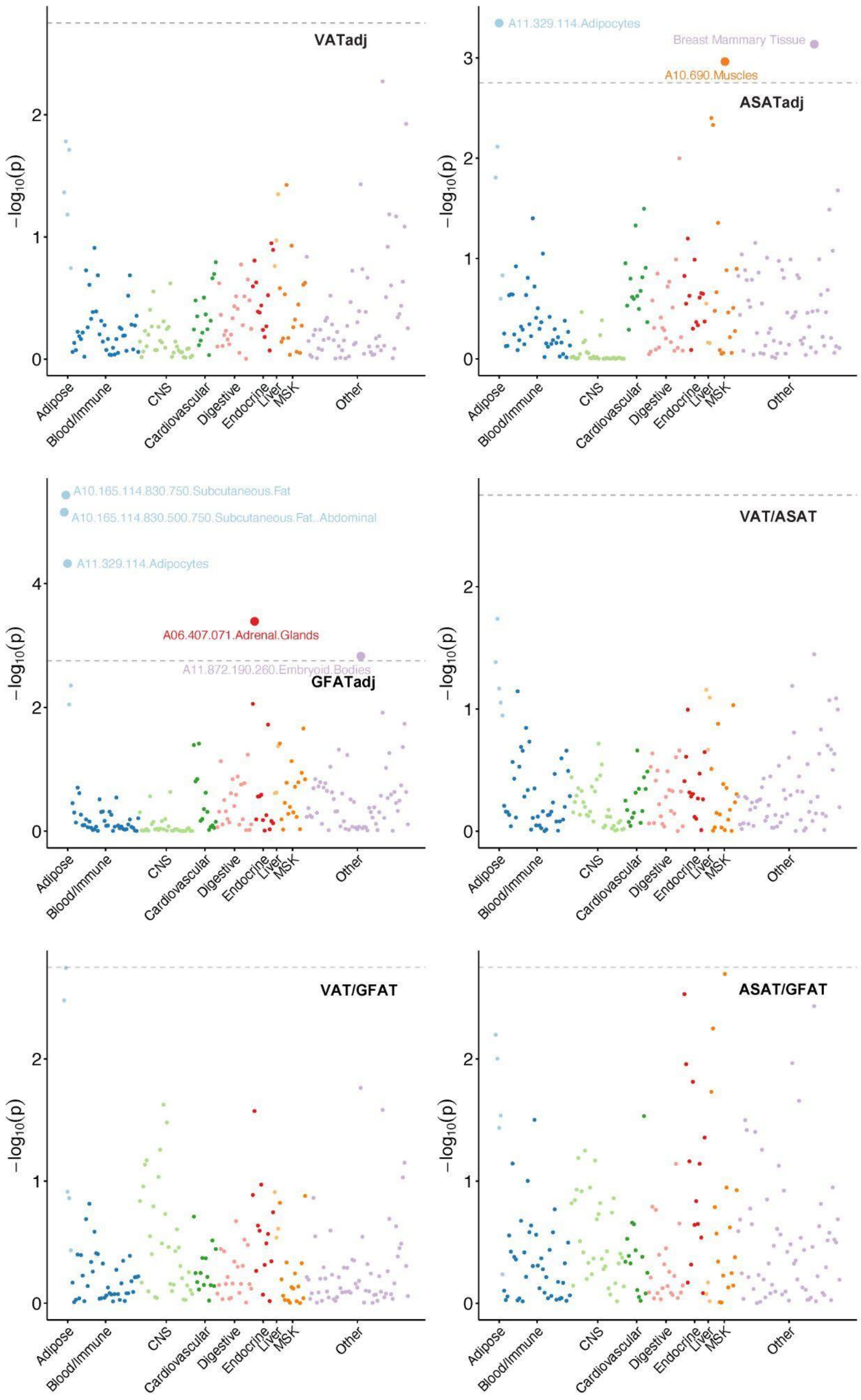

Supplementary Figure 17 Cell-type enrichment for local adiposity traits

Top left: VATadj; Top right: ASATadj, Middle left: GFATadj, Middle right: VAT/ASAT, Bottom left: VAT/GFAT, Bottom right: ASAT/GFAT. Each circle represents a tissue or cell type from either the GTEx dataset or the Franke lab dataset. Large circles pass the cutoff of FDR < 5% at –log10 (P) = 2.75.[17] Complete data tables corresponding to these plots are found in Supplementary Data 14.

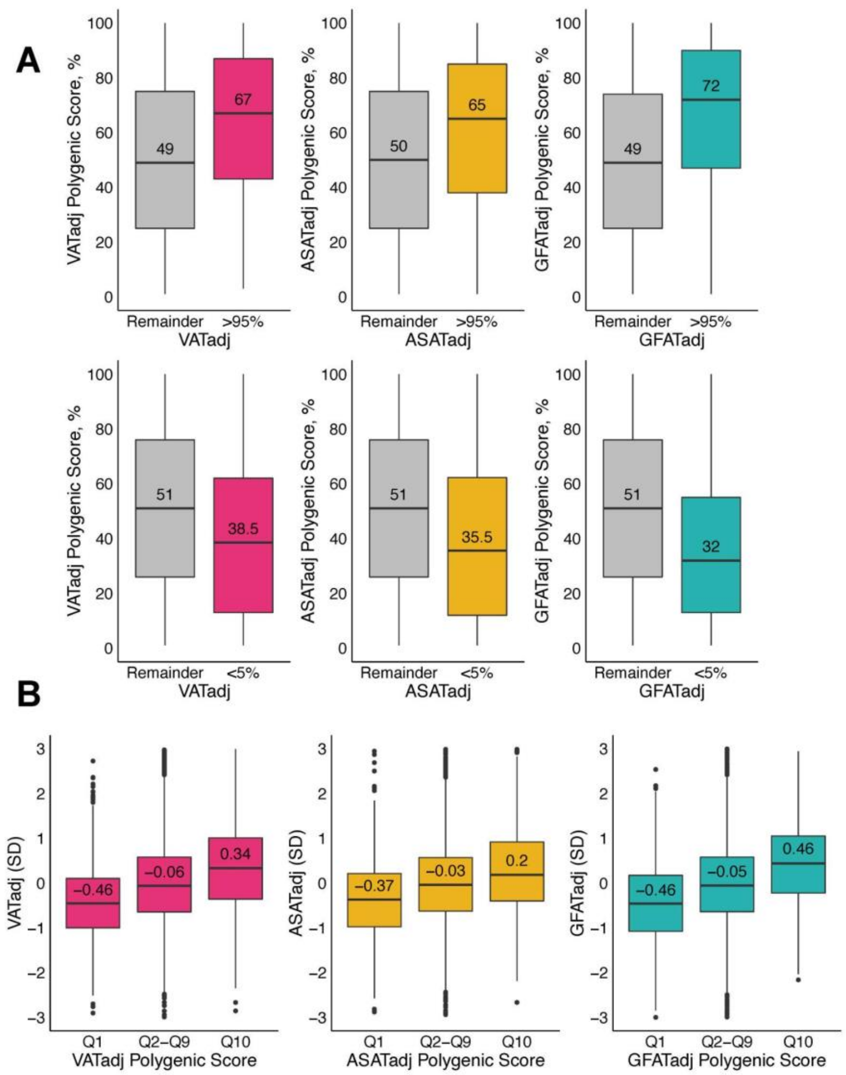

Supplementary Figure 18 Visualizing the relationship between VATadj, ASATadj, and GFATadj and their polygenic scores at the tails of the distributions

For each fat depot “adj” trait, a polygenic score was trained using LDpred2 on 70% of the studied cohort and a 10% validation cohort was used to determine the optimal set of hyperparameters. Results in this figure correspond to the 20% testing set (N = 7,795). (A) shows distribution of polygenic scores at the phenotypic tails of VATadj, ASATadj, and GFATadj. (B) shows distribution of VATadj, ASATadj, and GFATadj across deciles of the polygenic scores. Boxes contain median values and are bounded by the 1st and 3rd quartiles.

Supplementary Table 6 Heritability of adiposity phenotypes

	h_g^2 (BOLT-REML)			baselineLD model
**Phenotype**	**Combined**	**Males**	**Females**	**Combined**
VAT	0.310 (0.014)	0.296 (0.028)	0.401 (0.027)	0.194 (0.021)
ASAT	0.313 (0.014)	0.295 (0.028)	0.382 (0.027)	0.174 (0.023)
GFAT	0.360 (0.014)	0.332 (0.028)	0.422 (0.026)	0.207 (0.024)
VATadj	0.407 (0.015)	0.435 (0.029)	0.455 (0.027)	0.291 (0.027)
ASATadj	0.339 (0.015)	0.400 (0.029)	0.411 (0.027)	0.238 (0.024)
GFATadj	0.411 (0.015)	0.418 (0.029)	0.518 (0.027)	0.271 (0.028)
VAT/ASAT	0.407 (0.014)	0.453 (0.028)	0.430 (0.026)	0.288 (0.025)
VAT/GFAT	0.395 (0.014)	0.402 (0.028)	0.473 (0.026)	0.278 (0.022)
ASAT/GFAT	0.367 (0.014)	0.359 (0.028)	0.497 (0.026)	0.228 (0.023)
BMI	0.307 (0.015)	0.318 (0.029)	0.330 (0.028)	0.201 (0.024)
Waist circ.	0.248 (0.015)	0.229 (0.029)	0.297 (0.028)	0.140 (0.023)
WHR	0.216 (0.015)	0.223 (0.029)	0.275 (0.027)	0.128 (0.021)
WHRadjBMI	0.206 (0.014)	0.226 (0.028)	0.240 (0.027)	0.146 (0.021)

The first three columns are SNP-heritability estimates (h_g^2) obtained from BOLT-REML[18–20], while the fourth column contains heritability parameter estimates from LD-score regression with the baseline LD model.[21] On average, the heritability parameter estimate for the baselineLD model is 67% of the SNP-heritability estimates from BOLT-LMM, which is consistent with prior comparisons.[20] General trends include: (1) measures of local adiposity (adjusted-for-BMI and fat depot ratios) being more heritable than measures strongly correlated with global adiposity (BMI, VAT, ASAT, GFAT) and (2) most traits being more heritable in female participants (VAT/ASAT is the exception).

Supplementary Table 7 Nominally significant associations between the newly-identified adiposity loci in this study and cardiometabolic traits

Trait	CHR	BP	SNP	P-value	Nearest Gene	Nominally significant associations with cardiometabolic in the Type 2 Diabetes Knowledge Portal (P < 0.05)
GFAT	11	95840436	rs1074742	1.40E-08	MAML2	Assorted MAGIC insulin secretion during OGTT traits[22] (incremental insulin at 30 min OGTT, insulin at 30 min OGTT adjBMI, AUCins over AUCgluc), assorted IVGTT-based insulin secretion traits[23] (peak insulin response, acute insulin response), HbA1c adjBMI[24]
GFAT	12	124344710	rs138756410	3.00E-08	DNAH10	Obese vs. control OR Obese vs. thin[25], coronary artery disease[26], acute insulin response[23]
GFAT	12	125092343	rs4765159	3.50E-08	NCOR2	Waist circumference (+/- adj BMI-smoking status)[27,28], ratio total to HDL cholesterol, two-hour insulin
VATadj	2	121310704	rs35932591	3.80E-08	LINC01101	Triglcyerides[29,30], LDL-cholesterol[29,30], eGFR and BUN[31], Fasting insulin adjBMI[24], Systolic blood pressure[32], BMI[30], coronary artery disease[26], AST/ALT ratio[33], type 2 diabetes[34]**,** WHRadjBMI[16], HDL-cholesterol
VATadj	10	25767521	rs1329254	1.40E-08	GPR158	Diastolic blood pressure and systolic blood pressure[32], random blood glucose[29], BMI[16]
VATadj	11	69195097	rs7933253	1.30E-08	LOC102724265	WHRadjBMI[16], BMI[35], Hip circumference[8]
VATadj (Male)	2	121310704	rs35932591	3.90E-08	LINC01101	See entry for VATadj
VATadj (Female)	3	56901687	rs1500714	1.80E-08	ARHGEF3	Assorted MAGIC insulin secretion during OGTT traits[22] (AUC for insulin, insulin at 30 min OGTT, AUCins over AUCgluc, incremental insulin at 30 min OGTT, Matsuda insulin sensitivity index, corrected insulin response, insulin at 30 min OGTT adj BMI), WHRadjBMIsmoking and WaistadjBMIsmoking[28], TOAST small artery occlusion[36], ALT
ASATadj	1	201016296	rs3850625	1.80E-12	CACNA1S	eGFR[31], Diastolic blood pressure and systolic blood pressure[32], Fasting insulin adjBMI[24], Body fat percentage, AST/ALT ratio[33], WaistadjBMIsmoking[28], WaistadjBMI[8], Hip adjBMI[8], Leptin, BMI, coronary artery disease[26], HDL3 cholesterol[37], two-hour glucose adjBMI[24], Waist circumference, Controls vs. thin[25]
ASATadj	9	1044400	rs2048235	4.10E-08	LINC01230	Fasting insulin adjBMI[24], type 2 diabetes (or adjBMI)[38], AST/ALT ratio[33], ALT[33], coronary artery disease[26], body fat percentage, random blood glucose[29], eGFR-cys[39], obesity,
ASATadj	9	1052722	rs6474550	1.30E-09	DMRT2	AST/ALT ratio[33], Waist circumference (+/- adjBMI or adjBMIsmoking)[8,28], Triglycerides, Hip circumference (+/- adjBMI)[8], type 2 diabetes (+/- adjBMI)[38], BMIadjsmoking[28], WHR (+/- adjBMI)[8], Assorted MAGIC insulin secretion during OGTT traits[22] (AUC for insulin), ALT, BUN, eGFR-cys
ASATadj	15	62757857	rs17205757	3.20E-08	MIR6085	Pulse, systolic, and diastolic blood pressure[32], eGFR[31], LDL-cholesterol, BMI, Triglycerides, HbA1c, ALT, insulin sensitivity adjBMI, Obese vs. control[25], TOAST other determined, WHRadjBMI[16]
ASATadj	17	76324751	rs4444401	4.20E-08	SOCS3	Type 2 diabetes, AST[33], Assorted MAGIC insulin secretion during OGTT traits[22] (corrected insulin response), systolic and pulse blood pressure[32], HbA1cadjBMI[24], HDL-cholesterol, two-hour glucoseadjBMI[24], HipadjBMI[8]
ASATadj (Female)	1	116916645	rs749166380	2.20E-08	ATP1A1	Obese vs. control[25], trunk fat ratio[40]
ASATadj (Female)	8	58352327	rs776481989	8.60E-09	LOC101929488	
GFATadj	2	3648186	rs7588285	1.40E-08	COLEC11	LDL-cholesterol, triglycerides, total cholesterol, diastolic and systolic blood pressure[32], HDL-cholesterol, eGFR[31], obesity, coronary artery disease[26], AST/ALT ratio[33], Weight, Assorted MAGIC insulin secretion during OGTT traits[22] (Matsuda insulin sensitivity), Fasting insulin adjBMI[24]
GFATadj	2	226768344	2:226768344_CA_C	2.60E-08	NYAP2	
GFATadj	3	196818853	rs13099700	7.90E-09	DLG1	eGFR[31], WHRadjBMI (or WHR)[16], systolic and diastolic blood pressure[32], BMI, NAFLD in type 2 diabetes, Rankin stroke severity

GFATadj	5	38810354	rs142369482	9.10E-09	OSMR-AS1	Hypertension, waist circumference, weight
GFATadj	10	122970216	rs1907218	3.60E-10	FGFR2	Systolic, pulse, and diastolic blood pressure[32], type 2 diabetes (or adjBMI)[38], WHRadjBMI (or WHR or adjBMIsmoking)[16,28], AST/ALT ratio[33], Triglycerides, HDL-cholesterol, BMI, HipadjBMI[8], random glucose, Fasting insulin adjBMI[24], ALT
GFATadj (Male)	4	104780790	rs528845403	2.40E-08	TACR3	Arm fat ratio[40], Trunk fat ratio[40], Hypertension[41]
GFATadj (Female)	1	181161153	rs7550430	1.80E-09	LINC01732	Weight[42], hip circumference[42]
GFATadj (Female)	2	165533198	rs386652275	3.20E-08	COBLL1	
VAT/ASAT	2	178121005	rs13028464	4.80E-08	NFE2L2	eGFR or BUN[31], C-reactive protein, triglycerides, systolic, pulse, or diastolic blood pressure[32], LDL-cholesterol, WHRadjBMI[16], type 1 diabetes, TOAST other undetermined, stroke in type 2 diabetes, Arm fat ratio[40], Adiponectin, assorted IVGTT-based insulin secretion traits[23] (acute insulin response adj SI or adj BMI-SI), HDL-cholesterol, TOAST large artery atherosclerosis[36]
VAT/ASAT	6	19947871	rs70987287	1.70E-17	ID4	Ischemic stroke
VAT/ASAT	8	25459001	rs3890765	6.80E-09	CDCA2	WHRadjBMI (or WHR)[16], BUN, TOAST other undetermined, AST/ALT ratio[33], fasting plasma glucose[43]
VAT/ASAT	9	1054362	rs6474552	1.20E-08	DMRT2	AST/ALT ratio[33], Waist circumference (or adjBMI or adjBMIsmoking)[8,28], Triglycerides, Fasting insulin adjBMI[24], LDL-cholesterol, Assorted MAGIC insulin secretion during OGTT traits[22] (AUC for insulin, Matsuda insulin sensitivity), type 2 diabetes adjBMI[38], BUN, eGFR, Hip circumference[8], Obese vs. thin[25]
VAT/ASAT	10	63702572	rs55767272	6.80E-09	ARID5B	Triglycerides[29], WHR (or adjBMI)[16], BMI
VAT/ASAT	10	122992475	rs11199845	1.50E-14	FGFR2	Systolic, pulse, and diastolic blood pressure[32], type 2 diabetes (or adjBMI)[38], triglycerides[29], Fasting insulin adjBMI[24], BMI, AST/ALT ratio[33], coronary artery disease[26], random glucose, HDL-cholesterol[30]
VAT/ASAT (Male)	2	61760756	rs13390751	1.30E-08	XPO1	AST/ALT ratio[33], pulse and systolic blood pressure[32], BMI, LDL-cholesterol, triglycerides, coronary artery disease[26], ALT, total cholesterol, type 2 diabetes[38]
VAT/ASAT (Male)	6	19949170	6:19949170_GT_G	3.70E-09	ID4	
VAT/ASAT (Male)	10	122992442	rs11199844	5.90E-09	FGFR2	Systolic, pulse, and diastolic blood pressure[32], type 2 diabetes (or adjBMI)[38], Triglycerides[29], Fasting insulin adjBMI[24], BMI, AST/ALT ratio[33], coronary artery disease[26], HDL-cholesterol, random glucose, ALT
VAT/ASAT (Female)	6	19947871	rs70987287	8.50E-10	ID4	See entry for VAT/ASAT
VAT/ASAT (Female)	12	121319417	rs59757908	4.20E-08	SPPL3	HbA1c, pulse pressure
VAT/GFAT	14	94844947	rs28929474	4.80E-10	SERPINA1	AST, AST/ALT ratio, ALT, coronary artery disease, C-reactive protein, systolic, diastolic, and pulse blood pressure, type 2 diabetes (or adjBMI), trunk fat ratio and leg fat ratio, fasting insulin adjBMI, BMI, BUN, WHR (or adjBMI), triglycerides, total cholesterol, TOAST small artery occlusion, hip circumference, random glucose, serum ApoB, HbA1c adjBMI
VAT/GFAT (Female)	1	162430821	rs9660318	1.80E-08	UHMK1	ratio total to HDL cholesterol, HbA1c, TOAST other determined
VAT/GFAT (Female)	2	116072770	rs11399916	3.70E-08	DPP10	any cardiovascular disease[41]
VAT/GFAT (Female)	6	32975699	rs9276981	4.60E-08	HLA-DOA	type 1 diabetes[44], WHR (or adjBMI)[16], BMI, AST/ALT ratio[33]
ASAT/GFAT	5	55830865	rs39837	2.60E-08	LINC01948	AST/ALT ratio[33], WHR (or adjBMI)[16], type 2 diabetes adjBMI[38], LDL cholesterol, systolic and diastolic blood pressure[32], Fasting insulin adjBMI[24], HOMA-IR[45], coronary artery disease[26], eGFR, triglycerides, Stumvoll insulin sensitivity index[46], HDL3 cholesterol[37]
ASAT/GFAT	14	95219657	rs8006225	2.60E-09	GSC	WHRadjBMI (or WHR)[16], HbA1c adjBMI[24], systolic blood pressure[32], eGFR[31], TOAST small artery occlusion[36], HbA1c[47],

						two-hour glucose (or adjBMI)[48], coronary artery disease in type 2 diabetes[34], total cholesterol, hip circumference[8]
ASAT/GFAT	16	86424697	rs1552657	4.90E-08	LINC00917	Systolic, pulse, and diastolic blood pressure[32], triglycerides, LDL-cholesterol, Stumvoll insulin sensitivity index[46], eGFR[31], type 2 diabetes (or adjBMI)[38], arm fat ratio[40], Fasting insulin adjBMI[24], coronary artery disease[26]
ASAT/GFAT (Female)	5	55830865	rs39837	9.10E-09	LINC01948	See entry for ASAT/GFAT

All nominally significant associations with cardiometabolic traits ($P < 0.05$) were determined with the Type 2 Diabetes Knowledge Portal. In select cases where a large study made up most of the N for a given association, the individual study citation was included. Note that rs35932591 (VATadj and VATadj (Male)), rs70987287 (VAT/ASAT and VAT/ASAT (Female)), and rs39837 (ASAT/GFAT and ASAT/GFAT (Female)) are duplicated, so 39 unique lead SNPs are presented in this table. BP, GRCh37 position. P-value, BOLT-LMM association P-value.

Supplementary Table 8 Genomic inflation and LD-score intercepts

Phenotype (Combined)	λ_{GC} (Genomic inflation)	LD-score regression intercept
VAT	1.115	1.029 (0.007)
ASAT	1.110	1.025 (0.007)
GFAT	1.124	1.032 (0.008)
VATadj	1.136	1.031 (0.008)
ASATadj	1.125	1.026 (0.009)
GFATadj	1.137	1.050 (0.009)
VAT/ASAT	1.129	1.037 (0.008)
VAT/GFAT	1.135	1.032 (0.008)
ASAT/GFAT	1.138	1.028 (0.008)
**Phenotype (Males)**		
VAT	1.055	1.006 (0.007)
ASAT	1.059	1.019 (0.007)
GFAT	1.067	1.028 (0.007)
VATadj	1.077	1.010 (0.008)
ASATadj	1.079	1.021 (0.007)
GFATadj	1.077	1.031 (0.008)
VAT/ASAT	1.081	1.019 (0.007)
VAT/GFAT	1.072	1.005 (0.007)
ASAT/GFAT	1.061	1.017 (0.006)
**Phenotype (Females)**		
VAT	1.084	1.023 (0.006)
ASAT	1.082	1.019 (0.007)
GFAT	1.072	1.017 (0.008)
VATadj	1.069	1.024 (0.007)
ASATadj	1.090	1.023 (0.008)

GFATadj	1.104	1.031 (0.007)
VAT/ASAT	1.075	1.026 (0.007)
VAT/GFAT	1.090	1.026 (0.007)
ASAT/GFAT	1.109	1.030 (0.008)

Genomic inflation parameters (λ_{GC}) were computed from GWAS summary statistics including all directly genotyped and imputed SNPs. LD-score regression intercepts were computed using the original LD model with HapMap3 SNPs and default settings.[14]

Supplementary Table 9 Genetic correlations between adiposity traits in males and females

**Phenotype**	**Genetic correlation (r_g) between male and female summary statistics**
VAT	0.73 (0.09)
ASAT	0.90 (0.10)
GFAT	1.04 (0.11)
VATadj	0.87 (0.08)
ASATadj	0.80 (0.09)
GFATadj	0.79 (0.08)
VAT/ASAT	0.83 (0.08)
VAT/GFAT	0.70 (0.08)
ASAT/GFAT	0.80 (0.08)

Supplementary References

1. Agrawal S, Klarqvist MDR, Diamant N, et al. Association of machine learning-derived measures of body fat distribution in >40,000 individuals with cardiometabolic diseases. medRxiv 2021;2021.05.07.21256854.
2. Leinhard OD, Johansson A, Rydell J, et al. Quantitative abdominal fat estimation using MRI. In: 2008 19th International Conference on Pattern Recognition. 2008. p. 1–4.
3. Borga M, Thomas EL, Romu T, et al. Validation of a fast method for quantification of intra-abdominal and subcutaneous adipose tissue for large-scale human studies. NMR Biomed 2015;28(12):1747–53.
4. West J, Leinhard OD, Romu T, et al. Feasibility of MR-Based Body Composition Analysis in Large Scale Population Studies. PLOS ONE 2016;11(9):e0163332.
5. Borga M, West J, Bell JD, et al. Advanced body composition assessment: from body mass index to body composition profiling. J Investig Med Off Publ Am Fed Clin Res 2018;66(5):1–9.
6. Linge J, Borga M, West J, et al. Body Composition Profiling in the UK Biobank Imaging Study. Obes Silver Spring Md 2018;26(11):1785–95.
7. Linge J, Whitcher B, Borga M, Dahlqvist Leinhard O. Sub-phenotyping Metabolic Disorders Using Body Composition: An Individualized, Nonparametric Approach Utilizing Large Data Sets. Obes Silver Spring Md 2019;27(7):1190–9.
8. Shungin D, Winkler TW, Croteau-Chonka DC, et al. New genetic loci link adipose and insulin biology to body fat distribution. Nature 2015;518(7538):187–96.
9. Rüeger S, McDaid A, Kutalik Z. Evaluation and application of summary statistic imputation to discover new height-associated loci. PLoS Genet 2018;14(5):e1007371.
10. Kichaev G, Bhatia G, Loh P-R, et al. Leveraging Polygenic Functional Enrichment to Improve GWAS Power. Am J Hum Genet 2019;104(1):65–75.
11. Christakoudi S, Evangelou E, Riboli E, Tsilidis KK. GWAS of allometric body-shape indices in UK Biobank identifies loci suggesting associations with morphogenesis, organogenesis, adrenal cell renewal and cancer. Sci Rep 2021;11(1):10688.
12. Chu AY, Deng X, Fisher VA, et al. Multiethnic genome-wide meta-analysis of ectopic fat depots identifies loci associated with adipocyte development and differentiation. Nat Genet 2017;49(1):125–30.
13. Fox CS, White CC, Lohman K, et al. Genome-wide association of pericardial fat identifies a unique locus for ectopic fat. PLoS Genet 2012;8(5):e1002705.
14. Bulik-Sullivan BK, Loh P-R, Finucane HK, et al. LD Score regression distinguishes confounding from polygenicity in genome-wide association studies. Nat Genet 2015;47(3):291–5.
15. Bulik-Sullivan B, Finucane HK, Anttila V, et al. An atlas of genetic correlations across human diseases and traits. Nat Genet 2015;47(11):1236–41.
16. Pulit SL, Stoneman C, Morris AP, et al. Meta-analysis of genome-wide association studies for body fat distribution in 694 649 individuals of European ancestry. Hum Mol Genet 2019;28(1):166–74.
17. Finucane HK, Reshef YA, Anttila V, et al. Heritability enrichment of specifically expressed genes identifies disease-relevant tissues and cell types. Nat Genet 2018;50(4):621–9.
18. Loh P-R, Bhatia G, Gusev A, et al. Contrasting genetic architectures of schizophrenia and other complex diseases using fast variance-components analysis. Nat Genet 2015;47(12):1385–92.
19. Loh P-R, Tucker G, Bulik-Sullivan BK, et al. Efficient Bayesian mixed-model analysis increases association power in large cohorts. Nat Genet 2015;47(3):284–90.
20. Loh P-R, Kichaev G, Gazal S, Schoech AP, Price AL. Mixed-model association for biobank-scale datasets. Nat Genet 2018;50(7):906–8.

21. Gazal S, Finucane HK, Furlotte NA, et al. Linkage disequilibrium-dependent architecture of human complex traits shows action of negative selection. Nat Genet 2017;49(10):1421–7.
22. Prokopenko I, Poon W, Mägi R, et al. A central role for GRB10 in regulation of islet function in man. PLoS Genet 2014;10(4):e1004235.
23. Wood AR, Jonsson A, Jackson AU, et al. A Genome-Wide Association Study of IVGTT-Based Measures of First-Phase Insulin Secretion Refines the Underlying Physiology of Type 2 Diabetes Variants. Diabetes 2017;66(8):2296–309.
24. Chen J, Spracklen CN, Marenne G, et al. The trans-ancestral genomic architecture of glycemic traits. Nat Genet 2021;53(6):840–60.
25. Riveros-McKay F, Mistry V, Bounds R, et al. Genetic architecture of human thinness compared to severe obesity. PLoS Genet 2019;15(1):e1007603.
26. van der Harst P, Verweij N. Identification of 64 Novel Genetic Loci Provides an Expanded View on the Genetic Architecture of Coronary Artery Disease. Circ Res 2018;122(3):433–43.
27. Graff M, Scott RA, Justice AE, et al. Genome-wide physical activity interactions in adiposity - A meta-analysis of 200,452 adults. PLoS Genet 2017;13(4):e1006528.
28. Justice AE, Winkler TW, Feitosa MF, et al. Genome-wide meta-analysis of 241,258 adults accounting for smoking behaviour identifies novel loci for obesity traits. Nat Commun 2017;8:14977.
29. Forgetta V, Jiang L, Vulpescu NA, et al. An Effector Index to Predict Causal Genes at GWAS Loci [Internet]. 2021 [cited 2021 Nov 7]. Available from: https://www.biorxiv.org/content/10.1101/2020.06.28.171561v2
30. Kanai M, Akiyama M, Takahashi A, et al. Genetic analysis of quantitative traits in the Japanese population links cell types to complex human diseases. Nat Genet 2018;50(3):390–400.
31. Wuttke M, Li Y, Li M, et al. A catalog of genetic loci associated with kidney function from analyses of a million individuals. Nat Genet 2019;51(6):957–72.
32. Evangelou E, Warren HR, Mosen-Ansorena D, et al. Genetic analysis of over 1 million people identifies 535 new loci associated with blood pressure traits. Nat Genet 2018;50(10):1412–25.
33. Sinnott-Armstrong N, Tanigawa Y, Amar D, et al. Genetics of 35 blood and urine biomarkers in the UK Biobank. Nat Genet 2021;53(2):185–94.
34. Zhao W, Rasheed A, Tikkanen E, et al. Identification of new susceptibility loci for type 2 diabetes and shared etiological pathways with coronary heart disease. Nat Genet 2017;49(10):1450–7.
35. Yengo L, Sidorenko J, Kemper KE, et al. Meta-analysis of genome-wide association studies for height and body mass index in ~700000 individuals of European ancestry. Hum Mol Genet 2018;27(20):3641–9.
36. Malik R, Chauhan G, Traylor M, et al. Multiancestry genome-wide association study of 520,000 subjects identifies 32 loci associated with stroke and stroke subtypes. Nat Genet 2018;50(4):524–37.
37. Locke AE, Steinberg KM, Chiang CWK, et al. Exome sequencing of Finnish isolates enhances rare-variant association power. Nature 2019;572(7769):323–8.
38. Mahajan A, Taliun D, Thurner M, et al. Fine-mapping type 2 diabetes loci to single-variant resolution using high-density imputation and islet-specific epigenome maps. Nat Genet 2018;50(11):1505–13.
39. Gorski M, van der Most PJ, Teumer A, et al. 1000 Genomes-based meta-analysis identifies 10 novel loci for kidney function. Sci Rep 2017;7:45040.
40. Rask-Andersen M, Karlsson T, Ek WE, Johansson Å. Genome-wide association study of

body fat distribution identifies adiposity loci and sex-specific genetic effects. Nat Commun 2019;10(1):339.
41. Guindo-Martínez M, Amela R, Bonàs-Guarch S, et al. The impact of non-additive genetic associations on age-related complex diseases. Nat Commun 2021;12(1):2436.
42. Gurdasani D, Carstensen T, Fatumo S, et al. Uganda Genome Resource Enables Insights into Population History and Genomic Discovery in Africa. Cell 2019;179(4):984-1002.e36.
43. Nagy R, Boutin TS, Marten J, et al. Exploration of haplotype research consortium imputation for genome-wide association studies in 20,032 Generation Scotland participants. Genome Med 2017;9(1):23.
44. Robertson CC, Inshaw JRJ, Onengut-Gumuscu S, et al. Fine-mapping, trans-ancestral and genomic analyses identify causal variants, cells, genes and drug targets for type 1 diabetes. Nat Genet 2021;53(7):962–71.
45. Dupuis J, Langenberg C, Prokopenko I, et al. New genetic loci implicated in fasting glucose homeostasis and their impact on type 2 diabetes risk. Nat Genet 2010;42(2):105–16.
46. Walford GA, Gustafsson S, Rybin D, et al. Genome-Wide Association Study of the Modified Stumvoll Insulin Sensitivity Index Identifies BCL2 and FAM19A2 as Novel Insulin Sensitivity Loci. Diabetes 2016;65(10):3200–11.
47. Wheeler E, Leong A, Liu C-T, et al. Impact of common genetic determinants of Hemoglobin A1c on type 2 diabetes risk and diagnosis in ancestrally diverse populations: A transethnic genome-wide meta-analysis. PLoS Med 2017;14(9):e1002383.
48. Saxena R, Hivert M-F, Langenberg C, et al. Genetic variation in GIPR influences the glucose and insulin responses to an oral glucose challenge. Nat Genet 2010;42(2):142–8.
