# Supplementary material for: Inherited basis of visceral, abdominal subcutaneous and gluteofemoral fat depots
Source: Nat Commun. 2022 Jun 30;13:3771. doi: 10.1038/s41467-022-30931-2 (PMC9247093; doi:10.1038/s41467-022-30931-2)
Supplement: Supplementary file 3 — Description of Additional Supplementary Files [file 41467_2022_30931_MOESM3_ESM.pdf]

Description of Additional Supplementary Files

File name: Supplementary Data 1

Description: Baseline characteristics of genotyped cohort

File name: Supplementary Data 2

Description: Demographics and anthropometric traits differ between imaged and non-imaged individuals of the UK Biobank

File name: Supplementary Data 3

Description: Lead SNPs

File name: Supplementary Data 4

Description: Overlap of 250 lead SNPs in this study with traits relevant to adiposity in GWAS catalog

File name: Supplementary Data 5

Description: Association of ACVR1C missense variant rs56188432 with adiposity traits

File name: Supplementary Data 6

Description: Traits used for clustering gluteofemoral adiposity genetic loci

File name: Supplementary Data 7

Description: GFATadj clustering results

File name: Supplementary Data 8

Description: GFAT clustering results

File name: Supplementary Data 9

Description: GFATadj clustering results including VATadj and ASATadj

File name: Supplementary Data 10

Description: Sex heterogeneity analysis

File name: Supplementary Data 11

Description: Associating WHRadjBMI loci with VATadjBMI, ASATadjBMI, and GFATadjBMI

File name: Supplementary Data 12

Description: External validation of VAT, ASAT, VATadj, and VAT/ASAT genome-wide significant loci

File name: Supplementary Data 13

Description: Transcriptome-wide association study results

File name: Supplementary Data 14

Description: Cell-type enrichment analyses for nine adiposity traits in this study

File name: Supplementary Data 15

Description: Significant rare variant association study results

File name: Supplementary Data 16

Description: Focused investigation of PDE3B, ACAT1, and PCSK1 rare variant carriers

File name: Supplementary Data 17

Description: Focused investigation of PPARG and LMNA rare variant carriers

File name: Supplementary Data 18

Description: Association of candidate polygenic scores with corresponding adiposity trait

File name: Supplementary Data 19

Description: Variance in adiposity traits explained by genome-wide polygenic scores

File name: Supplementary Data 20

Description: Effects of VATadj, ASATadj, and GFATadj polygenic scores on metabolically relevant biomarkers and diseases in non-imaged subset of UK Biobank

File name: Supplementary Data 21

Description: Effects of VATadj, ASATadj, and GFATadj polygenic scores on selected traits in ARIC

File name: Supplementary Data 22

Description: Adjusted fat depots and collider bias
