# Supplementary material for: Inherited basis of visceral, abdominal subcutaneous and gluteofemoral fat depots
Source: Nat Commun. 2022 Jun 30;13:3771. doi: 10.1038/s41467-022-30931-2 (PMC9247093; doi:10.1038/s41467-022-30931-2)
Supplement: Supplementary file 5 — Reporting Summary [file 41467_2022_30931_MOESM5_ESM.pdf]

nature portfolio

Corresponding author(s): Saaket Agrawal, Amit V. Khera

Last updated by author(s): Apr 11, 2022

Reporting Summary

Nature Portfolio wishes to improve the reproducibility of the work that we publish. This form provides structure for consistency and transparency in reporting. For further information on Nature Portfolio policies, see our Editorial Policies and the Editorial Policy Checklist.

Statistics

For all statistical analyses, confirm that the following items are present in the figure legend, table legend, main text, or Methods section.

n/a	Confirmed	
☐	☒	The exact sample size (*n*) for each experimental group/condition, given as a discrete number and unit of measurement
☐	☒	A statement on whether measurements were taken from distinct samples or whether the same sample was measured repeatedly
☐	☒	The statistical test(s) used AND whether they are one- or two-sided<br>*Only common tests should be described solely by name; describe more complex techniques in the Methods section.*
☐	☒	A description of all covariates tested
☐	☒	A description of any assumptions or corrections, such as tests of normality and adjustment for multiple comparisons
☐	☒	A full description of the statistical parameters including central tendency (e.g. means) or other basic estimates (e.g. regression coefficient) AND variation (e.g. standard deviation) or associated estimates of uncertainty (e.g. confidence intervals)
☐	☒	For null hypothesis testing, the test statistic (e.g. *F*, *t*, *r*) with confidence intervals, effect sizes, degrees of freedom and *P* value noted<br>*Give P values as exact values whenever suitable.*
☒	☐	For Bayesian analysis, information on the choice of priors and Markov chain Monte Carlo settings
☒	☐	For hierarchical and complex designs, identification of the appropriate level for tests and full reporting of outcomes
☐	☒	Estimates of effect sizes (e.g. Cohen's *d*, Pearson's *r*), indicating how they were calculated

Our web collection on statistics for biologists contains articles on many of the points above.

Software and code

Policy information about availability of computer code

Data collection	All data were provided by the UK Biobank. No software was used for data collection.
Data analysis	Genome-wide association analyses and SNP-heritability calculations were done with BOLT-LMM v2.3.4. Genetic correlations between traits and cell- and tissue-specific enrichment analyses were done with ldsc v1.0.1. Lead SNPs were prioritized with LD clumping uisng PLINK v1.9. Identification of sex-dimorphic signals was done with EasyStrata v8.6. Transcriptome-wide association studies were conducted with the most recent release of FUSION as of July 2021. Rare variants were annotated with the Loss-of-Function Transcript Effect Estimator (LOFTEE) v1.0.2 algorithm implemented within the Ensembl Variant Effect Predictor (VEP) software. Polygenic score weights were computed using LDpred2 implemented in bigsnpr v1.7.1. All statistical analyses were done in R 3.6.0.

For manuscripts utilizing custom algorithms or software that are central to the research but not yet described in published literature, software must be made available to editors and reviewers. We strongly encourage code deposition in a community repository (e.g. GitHub). See the Nature Portfolio guidelines for submitting code & software for further information.

Data

Policy information about availability of data

All manuscripts must include a data availability statement. This statement should provide the following information, where applicable:

- Accession codes, unique identifiers, or web links for publicly available datasets
- A description of any restrictions on data availability
- For clinical datasets or third party data, please ensure that the statement adheres to our policy

This research has been conducted using the UK Biobank Resource under Application Number #7089. The raw UK Biobank data is made available to researchers from universities and other research institutions with genuine research inquiries, following IRB and UK Biobank approval. The GWAS summary statistics and polygenic

score weights generated in this study will be made available for download from the Cardiovascular Disease Knowledge Portal under the "Downloads" tab at https://cvd.hugeamp.org/ at the time of publication. All other relevant results are available in the supplemental information.

Field-specific reporting

Please select the one below that is the best fit for your research. If you are not sure, read the appropriate sections before making your selection.

- [x] Life sciences
- [ ] Behavioural & social sciences
- [ ] Ecological, evolutionary & environmental sciences

For a reference copy of the document with all sections, see nature.com/documents/nr-reporting-summary-flat.pdf

Life sciences study design

All studies must disclose on these points even when the disclosure is negative.

Sample size	The flow diagram in Supplementary Figure 1 how up to 43,531 individuals with MRI images were available in the UK Biobank. After imaging QC and taking individuals who were genotyped, 39,076 remained. After participant genetic QC, up to 38,965 individuals were included in genome-wide association analyses. These sample sizes were sufficient to enable discovery of new genetic loci associated with fat distribution at a genome-wide significance threshold of 5E-08.
Data exclusions	Imaging level QC was done on the basis of several criteria including fat/water swaps, individuals being misaligned, and individuals being too large for the scanner -- these are described fully in another manuscript that is cited by this paper (https://www.medrxiv.org/content/10.1101/2021.05.07.21256854v1). Participants were then excluded from genetic analyses if they met any of the following criteria: (1) mismatch between self-reported sex and sex chromosome count, (2) sex chromosome aneuploidy, (3) genotyping call rate <0.95, or (4) were outliers for heterozygosity.
Replication	Polygenic scores derived from GWAS summary statistics of VATadj, ASATadj, and GFATadj were associated with anthropometric and metabolic traits in a 20% held out testing set among imaged individuals, and further validated in an independent dataset of 447,486 individuals of the UK Biobank who were genotyped, but not imaged. All code for the results reported in the manuscript were run at least twice and internally consistent.
Randomization	Samples were not randomized. Genome-wide association analyses were adjusted for age at the time of imaging, age squared, sex, the top 10 principal components of genetic ancestry, MRI imaging center, and genotyping array.
Blinding	The machine learning model used to estimate VAT, ASAT, and GFAT volumes (https://www.medrxiv.org/content/10.1101/2021.05.07.21256854v1) was built without knowledge of phenotype or genetic associations. Polygenic scores extracted were blinded to the phenotype status of participants.

Reporting for specific materials, systems and methods

We require information from authors about some types of materials, experimental systems and methods used in many studies. Here, indicate whether each material, system or method listed is relevant to your study. If you are not sure if a list item applies to your research, read the appropriate section before selecting a response.

Materials & experimental systems

n/a	Involved in the study	
x		Antibodies
x		Eukaryotic cell lines
x		Palaeontology and archaeology
x		Animals and other organisms
	x	Human research participants
x		Clinical data
x		Dual use research of concern

Methods

n/a	Involved in the study	
x		ChIP-seq
x		Flow cytometry
x		MRI-based neuroimaging

Human research participants

Policy information about studies involving human research participants

Population characteristics	As described in Supplementary Table S1, mean age was 64.5 years, 51% were female, and 97% were white. Mean body mass index for both males (27.1) and females (26.0) was in the overweight category.
Recruitment	The UK Biobank is an observational study that enrolled over 500,000 individuals between the ages of 40 and 69 years between 2006 and 2010 (via mailer recruitment), of whom 43,521 underwent MRI imaging between 2014 and 2020.
Ethics oversight	Mass General Brigham institutional review board; UK Biobank application #7089.

Note that full information on the approval of the study protocol must also be provided in the manuscript.
